# CDKL5 sculpts functional callosal connectivity to promote cognitive flexibility

**DOI:** 10.1038/s41380-023-01962-y

**Published:** 2023-02-03

**Authors:** Patricia Nora Awad, Valerio Zerbi, Erin M. Johnson-Venkatesh, Francesca Damiani, Marco Pagani, Marija Markicevic, Sarah Nickles, Alessandro Gozzi, Hisashi Umemori, Michela Fagiolini

**Affiliations:** 1 F. M. Kirby Neurobiology Center, Department of Neurology, Boston Children’s Hospital, Harvard Medical School, Boston, MA USA; 2 Neural Control of Movement Lab, Department of Health Sciences and Technology, ETH Zurich, Zurich, Switzerland; 3 Neuro-X Institute, School of Engineering (STI), École Polytechnique Fédérale de Lausanne, Lausanne, Switzerland; 4 CIBM Center for Biomedical Imaging, Lausanne, Switzerland; 5 Functional Neuroimaging Laboratory, Center for Neuroscience and Cognitive Systems, Istituto Italiano di Tecnologia, Rovereto, Italy; 6 Autism Center, Child Mind Institute, New York, NY USA; 7 Hock E. Tan and K. Lisa Yang Center for Autism Research at Harvard University, Boston, MA USA; 8 International Research Center for Neurointelligence (IRCN), University of Tokyo Institutes for Advanced Study, Tokyo, Japan

**Keywords:** Neuroscience, Autism spectrum disorders

## Abstract

Functional and structural connectivity alterations in short- and long-range projections have been reported across neurodevelopmental disorders (NDD). Interhemispheric callosal projection neurons (CPN) represent one of the major long-range projections in the brain, which are particularly important for higher-order cognitive function and flexibility. However, whether a causal relationship exists between interhemispheric connectivity alterations and cognitive deficits in NDD remains elusive. Here, we focused on CDKL5 Deficiency Disorder (CDD), a severe neurodevelopmental disorder caused by mutations in the X-linked Cyclin-dependent kinase-like 5 (*CDKL5*) gene. We found an increase in homotopic interhemispheric connectivity and functional hyperconnectivity across higher cognitive areas in adult male and female CDKL5-deficient mice by resting-state functional MRI (rs-fMRI) analysis. This was accompanied by an increase in the number of callosal synaptic inputs but decrease in local synaptic connectivity in the cingulate cortex of juvenile CDKL5-deficient mice, suggesting an impairment in excitatory synapse development and a differential role of CDKL5 across excitatory neuron subtypes. These deficits were associated with significant cognitive impairments in CDKL5 KO mice. Selective deletion of CDKL5 in the largest subtype of CPN likewise resulted in an increase of functional callosal inputs, without however significantly altering intracortical cingulate networks. Notably, such callosal-specific changes were sufficient to cause cognitive deficits. Finally, when CDKL5 was selectively re-expressed only in this CPN subtype, in otherwise CDKL5-deficient mice, it was sufficient to prevent the cognitive impairments of CDKL5 mutants. Together, these results reveal a novel role of CDKL5 by demonstrating that it is both necessary and sufficient for proper CPN connectivity and cognitive function and flexibility, and further validates a causal relationship between CPN dysfunction and cognitive impairment in a model of NDD.

## Introduction

Miswiring of long- and short-range connectivity of neuronal networks has increasingly been reported in neurodevelopmental disorders (NDD) [1–4]. Interhemispheric connectivity represents a predominant type of long-range connection mediated by callosal projection neurons (CPN) whose axons bundle to form the corpus callosum (CC). They mediate hemispheric integration of lateralized cues and facilitate the execution of complex cortical functions that require bilateral processing, including cognition, social communication, visual and sensory-motor processing, and associative integration [5–8], all of which are commonly disrupted in NDD. Although NDD encompass a heterogenous group of individuals, there is evidence that interhemispheric connectivity is altered both at the level of structural integrity and functional connectivity (FC). Specifically, structural deficits of the CC have been found in patients with autism spectrum disorder (ASD) [9–12] and epilepsy [13–15] and associated mice models [16–19]. A growing body of work supports  that FC is also disrupted in NDD [13, 20–23], in fact two studies examining large cohorts of children with ASD (ABIDE I and II) report abnormalities of interhemispheric FC [21], and found evidence of hyperconnectivity in the anterior cingulate/medial prefrontal cortex (ACC/mPFC) [24]. Most importantly, the degree of disrupted interhemispheric connectivity correlates with ASD severity [20, 22, 24, 25]. Accordingly, the more severe condition of agenesis of the CC is also associated with cognitive and neurological impairments [26] and altered functional connectivity [27]. Mechanistically, NDD-related genes might be specifically involved in the development of CPN circuitry, as recently proposed for the ASD-risk gene FMR1 [28]. Together these findings raise the hypothesis that miswiring of interhemispheric connectivity may significantly contribute to network and behavioral abnormalities found in NDD. However, whether a causal mechanistic link exists between callosal dysfunction and disrupted behavioral output in NDD has not yet been directly investigated.

To tackle this question, we used a model of CDKL5 Deficiency Disorder (CDD), a rare developmental epileptic encephalopathy caused by de novo mutations on the X-linked autism-related Cyclin-dependent kinase-like 5 (*CDKL5*) gene affecting female and male individuals. CDD is characterized by intractable early-onset seizures, severe intellectual and language disability, autistic features as well as extensive cortical visual and motor impairments [29–33]. We reasoned that such severe cognitive deficits might be caused, at least partially, by callosal dysfunction. The *CDKL5* gene encodes a serine/threonine kinase that is highly expressed in early postnatal development and through adulthood [34]. Its expression is highest in layers 2/3 pyramidal neurons in the cortex and hippocampus (as illustrated in the Genotype-Tissue Expression GTEx database and Allen Brain Institute RNA dataset), overlapping with the major subtype of CPN. CDKL5 has been implicated in the activity-dependent regulation of synaptic and dendritic development, microtubule dynamics, and neuronal polarization [35, 36]. In this study, we explored whether the loss of CDKL5 disrupts long-range connectivity of CPN and mediates cognitive deficits in a rodent model of CDD that faithfully recapitulates the human phenotypes [37–41]. We combined a multi-level approach and identified functional hyperconnectivity across hemispheres and across higher cognitive cortical areas by resting-state functional MRI (rs-fMRI) analysis, and an increase in the number of callosal synaptic inputs in the cingulate cortex of CDKL5-deficient mice. Remarkably, selective deletion of CDKL5 in CPN recapitulated the cognitive deficits present in CDKL5 knockout (KO) mice; while expressing CDKL5 only in those neurons, in an otherwise knockout model, was sufficient to prevent cognitive function impairment. This study reveals a novel role of CDKL5 in long-range callosal maturation and points to a causal link between functional callosal overconnectivity and cognitive impairment in NDD.

## Materials and methods

### Animals

Animal care and experimental procedures were performed in accordance with the Institutional Animal Care and Use Committee (IACUC) of Boston Children’s Hospital. CDKL5^-/+^ (021967), C57BL/6J (000664), CDKL5^fl/fl^ (030523) and Satb2^Cre/+^ (030546) breeders were purchased from Jackson Laboratories. CDKL5 FloxStop line was kindly provided by Dr. Zhou [42]. Mice were raised on a 12 h light/dark cycle with food and water ad libitum, unless specified. Control animals were age- and sex-matched wild-type littermates. Experiments and data analysis were conducted blind to genotype.

### Magnetic resonance imaging

#### Data acquisition, preprocessing, and analysis

In vivo rs-fMRI data acquisition was performed using a Biospec 70/16 small animal MR system (Bruker BioSpin MRI, Ettlingen, Germany) and Paravision v6.1 as previously described [43]. Scans were obtained with a cryogenic quadrature surface coil (Bruker BioSpin AG, Fällanden, Switzerland). After common standard adjustments and anatomical images acquisition, a standard gradient-echo echo planar imaging sequence was used to acquire 900 volumes.

Resting state fMRI datasets were preprocessed using an existing pipeline [43] with modifications [44]. Thereafter, datasets were de-spiked, band-pass filtered (0.01–0.25 Hz), normalized first to an EPI study-specific template and then to the Allen Brain Institute reference atlas using ANTs v2.1. BOLD time series from 38 cortical ROIs in both hemispheres were extracted using the Allen Reference Atlas ontology. For cortical connectivity analysis, we calculated the cortical connectome (38 × 38 ROIs) in all animals by using Z-scored Pearson’s correlations and then quantified deviations between groups. To assess differences in connectivity profiles on a node-level, the effect size in/out of a given anatomic structure was summed. For our network analysis, we measured functional connectivity across 16 independent resting-state networks based on previous work [16]. We estimated a surrogate measure of network coupling strength using a dual regression approach [45].

#### Ex vivo diffusion tensor imaging

MRI-based diffusion-weighted (DW) imaging was carried out in PFA-fixed specimens as previously described [46]. DW imaging was performed using a 72-mm birdcage transmit coil and a saddle-shaped solenoid coil for signal reception [47].

Tract-based spatial statistics analysis was implemented in FSL [48]. Datasets were corrected for eddy current distortions and skull-stripped to remove extra-brain tissues. Voxelwise fractional anisotropy (FA) was calculated, and FA maps were nonlinearly registered to an in-house FA template with FLIRT and FNIRT and thinned using a FA threshold of 0.2 to create a skeleton of the white matter. Voxelwise intergroup comparison of FA was carried by using 5000 permutations (*p*  <  0.05, two-tailed). FA was also regionally quantified in 3 × 3 × 1 ROI placed to probe major white matter structures.

### Immunohistochemistry and imaging

Mice were transcardially perfused with 4% PFA (Sigma, cat #441244). Brains were sectioned with a cryostat (Leica CM3050 S) and incubated in the following primary antibodies: anti-VGlut1 (Synaptic Systems cat#135 303, 1:1000), anti-Vglut2 (Synaptic Systems cat#135 404, 1:2000), anti-Myelin Basic Protein (Millipore MAB384, 1:150). Sections were counterstained with DAPI (300 nM, Invitrogen D3571).

Images were acquired with a laser scanning confocal microscope (Zeiss 710) using a ×63 objective (1.4 NA, VGlut) or ×20 objective (0.8 NA, MBP). Quantitative analyses were performed on a minimum of 4–6 sections per mouse, in 3–6 mice per genotype. Analysis was performed with ImageJ software.

### Slice preparation and electrophysiology

We evaluated fiber fraction and miniature EPSCs as previously described [49]. Cortical sections were kept in artificial cerebral spinal fluid (ACSF) containing (mM): 118NaCl, 2.5 KCl, 1.3 MgCl_2_, 1.2 NaH_2_PO_4_, 2.5 CaCl_2_, 10 glucose, and 26 NaHCO_3_. Data was obtained with a Multiclamp 700B amplifier (Axon Instruments), digitized with Digidata 1440A (Axon Instruments) and collected with Clampex 10.7 (Axon Instruments). The internal solution for mEPSC recording contained (mM): 100 Cs-gluconate, 0.2 EGTA, 5 MgCl_2_, 2 Mg ATP, 0.3 Li GTP, and 40 HEPES, pH adjusted to 7.2 with CsOH. The solution for fiber fraction recordings contained (mM): 35 CsF, 100 CsCl_2_, 10 EGTA, and 10 HEPES, pH adjusted to 7.3 with CsOH. ACSF was supplemented during recording with 500 nM tetrodotoxin (mEPSCs) and 50 µM picrotoxin. For stimulation experiments, a concentric bipolar electrode (KO; FHC, Inc.) or a pair of glass electrodes, filled with 1 M NaCl and 25 mM HEPES (cKO, Het), were placed in the CC and responses were evoked using a A365 Stimulus Isolator (WPI) set between 0.1 and 1 mA. Minimal callosal responses were determined using the failure method (response <50%). The maximal response was the largest evoked amplitude which reached a plateau for three consecutive increases in stimulation intensity. Fiber fraction responses and mEPSCs were analyzed with Clampfit 10.7 (Axon) and Minianalysis (Synaptosoft) respectively.

### In vitro voltage-sensitive dye Imaging

We performed voltage-sensitive dye imaging (VSDI) as previously described [50]. Brains were kept in ACSF (containing (in mM): 130 NaCl, 10 glucose, 24 NaHCO_3_, 3.5 KCl, 1.25 NaH_2_PO_4_, 2.5 CaCl_2_ and 1.5 MgCl_2_), and slices incubated in the dye Di-4-ANEPPS (Invitrogen; D-1199; 5 μg/ml). Fibers were stimulated (0.1–1 mA, 1 ms pulse) with an ACSF-filled glass pipette and delivered with a constant current stimulus isolator (Iso-Flex, A.M.P.I., different for females). Excitation light source was a LED illumination system (530 nm wavelength, LEX3-G, SciMedia). Emitted fluorescence was long-pass filtered (590 nm) and imaged with a MiCam Ultima (SciMedia; 1-ms frame rate; 512-ms period). Time course traces were averaged across ten trials and exported to BV Ana software (SciMedia) for analysis. Fluorescence change was normalized to resting fluorescence (∆*F*/*F*).

### Behavior testing

#### Olfactory habituation/dishabituation

Olfactory discrimination was performed in a static cage, where a cotton swab was hanging from the cage cover. Following habituation to the cage and cotton swab, odors were dipped in the following scents: water, anise (1:100 in ddH_2_0) and clove (1:100 in ddH_2_0). Three trials of 2 min each were performed, and time spent sniffing were manually recorded.

#### Four-choice foraging task

Four-choice task was performed as previously described [51]. Odor stimuli consisted of wood shavings (Kaytee aspen bedding, Chilton, WI) scented with odor cues (anise extract (McCormick, MD) clove, litsea and eucalyptus oils (1:10 in mineral oil; San Francisco Massage Supply Co., CA); and thymol (1:20 in 50% ethanol, Alfa Aesar, cat#A14563; mixed with shaving at 0.02 ml/g). Mice were placed on a restricted diet for 3 days prior to the start (~90% weight). The location of the pot with the food reward (~10 mg Cheerios) was pseudo-randomized. Criterion for each task was eight correct digging choices out of ten consecutives trials, to a maximum of 120 trials. Two mice were unable to stay awake during the whole test and were excluded from the analysis. Omission trials where no choice was made were analyzed separately.

#### Morris water maze

Morris water maze was run on 3–5 months old mice as previously described [52, 53]. All swim patterns were recorded and analyzed with EthoVision XT (Noldus). Mice who simply floated were excluded from analysis.

### Statistical analysis

All data are presented as mean ± standard error, with n and ages described in figure legends. Sample sizes for each experiment was decided based on our prior work, and power analysis conducted with a false-positive rate of 0.05 and the desired power of 95%. Each experiment included WT and mutant mice from multiple litters. Normality distribution of the data was assessed by Shapiro-Wilk normality test. To compare between two groups, we performed the parametric unpaired two-tailed *t* test, or the nonparametric Mann-Whitney test (FDR-corrected for DTI or corrected for multiple comparison using Holm-Sidak method). Statistical tests on repeated measures were conducted using two-way ANOVA or Mixed model analyses, with Sidak correction for multiple comparisons. Outliers were identified by the ROUT method (*Q* = 0.1%). Statistical tests were performed using Prism (v9.4). The statistical significance was set at *p* < 0.05.

### Reporting summary

Further information on research design is available in the Nature Portfolio Reporting Summary linked to this article. Additional detailed methods can be found in supplementary material.

## Results

### Functional hyperconnectivity in adult CDKL5 KO mice

**Fig. 1:**
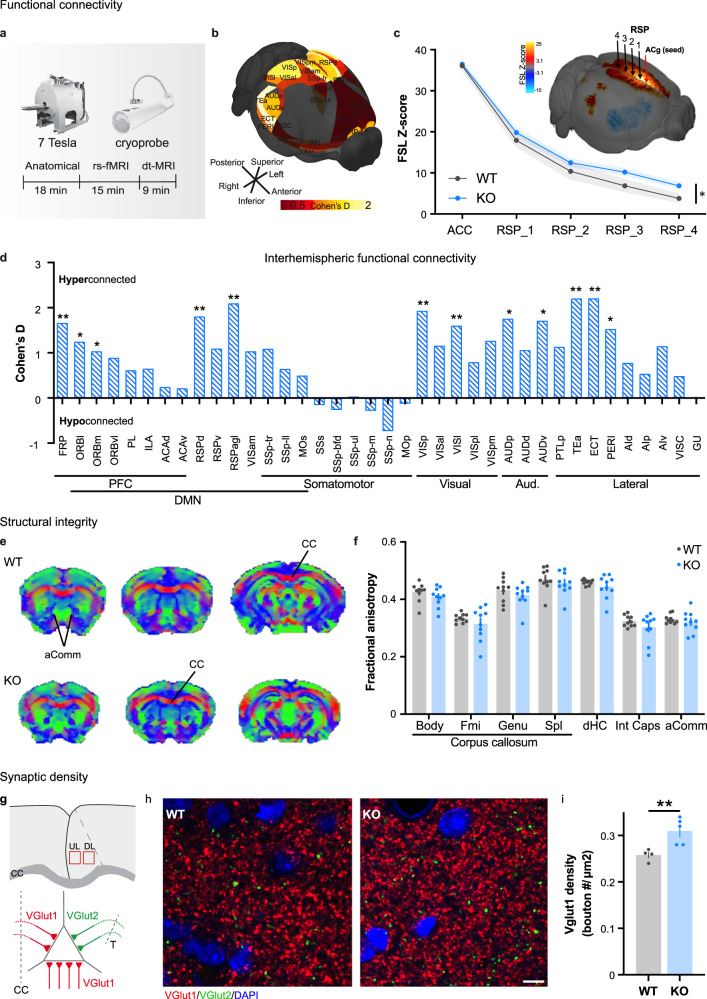
Functional connectivity and anatomical alterations in adult CDKL5 KO mice. **a** Schematic representation of the experimental procedure of functional imaging. **b** 3D anatomical representation of unbiased connectional alterations in the CDKL5 KO compared to its WT littermates. **c** (Top) 3D visual representation of seed-based analysis originating from the cingulate cortex. (Bottom) Connectivity coupling was measured using Z-scored normalized Pearson’s correlation coefficient (FSLNets; two-way Anova, region *F*_4,70_ = 182.1, *p* < 0.0001, genotype: *F*_1,70_ = 6.873, *p* = 0.0107*). **d** Cohen D’s effect size of interhemispheric functional connectivity evaluated in different brain regions between WT and KO mice. (*n* = 10/group, multiple t-test, Bonferonni corrected). **e** Fractional anisotropy (FA) brain maps in a representative WT (top) and KO (bottom) mouse. Images are color coded by the first eigenvector of the FA, red for the left-right, green for anterior-posterior and blue for top-bottom direction. **f** Quantification of FA in the anterior commissure, CC, dorsal hippocampus, and internal capsule as assessed with diffusion-weighted imaging. Intergroup comparisons showed preserved FA in KO mutant mice with respect to control littermates (multiple t-test FDR corrected, *p* > 0.05 all regions). CC Body, body of the CC; CC Fmi, forcep minor of the CC; CC Genu, genu of the CC; CC Spl, splenium of the CC. *n* = 10 mice/group. **g** (Top) Schematic of region of interest in the cingulate cortex, in layers 2–4 and deeper layers (L5–6). (Bottom) Schematic representation of VGlut1 staining labeling both callosal and intracortical synapses (red), while VGlut2 labels excitatory thalamo-cortical synapses (green, CC: Corpus Callosum, T: Thalamus). **h** Example images of glutamatergic transporter markers VGlut1 (red), VGlut 2 (green) and DAPI (blue) staining. Scale bar, 5 µm. **i** VGlut1 density in all layers (WT: 0.262 ± 0.007, *n* = 4 mice (24 sections), KO: 0.318 ± 0.011, *n* = 5mice (29 section), Mann-Whitney test *p* = 0.0079**) Mean ± SEM.

In order to evaluate whole-brain functional connectivity in the total absence of CDKL5, we performed rs-fMRI analysis in adult CDKL5^-/y^ compared to CDKL5^+/y^ mice (hereon referred to as KO and WT respectively; Fig. 1a). The 3D brain schematic in Fig. 1b illustrates inter-group differences of connectivity strengths during resting state as a heatmap. We identified that FC across posterior sensory cortical areas and components of PFC and default mode network (DMN) [54] were hyper-synchronized in the KO mice compared to WT littermates (Fig. 1b, Suppl. Fig 1a, b). This was further confirmed by independent component analysis (ICA, Suppl. Fig. 1c-g). We investigated the FC of the anterior cingulate cortex (ACC) in particular as it is an essential hub in multiple brain-wide networks relevant to the expression of complex behaviors and higher order cognitive function such as cognitive flexibility, reinforcement learning, attention, and emotion processing [55, 56]. A seed-voxel analysis revealed a significant hyperconnectivity between the ACC and the retrosplenial cortex (RSP, Fig. 1c, *p* = 0.005). To specifically evaluate long-range interhemispheric homotopic connectivity, we compared the mean cortical connectivity strength across hemispheres between WT and KO as Cohen’s D effect size valuations (Fig. 1d). We found a significant increase in interhemispheric connectivity in KO mice, in regions particularly involving the DMN and sensory cortices. We further confirmed these connectivity changes in a separate cohort of heterozygous CDKL5 females (hereon referred to as Het), in light of the prevalence of the disease in female patients. We found a similar increase in connectivity between the ACC and RSP compared to their sex-matched controls (Suppl. Fig. 2a). Interhemispheric connectivity was also evaluated and while several areas trended in the same manner as in the KO males, only the frontal cortex and motor cortex were shown to be significantly overconnected (Suppl. Fig. 2b). These results revealed that homotopic connectivity, as well as PFC-dependent networks were increased in CDKL5-deficient mice.

To investigate whether the increase in functional connectivity in CDKL5 KO mice could be related to meso- or microstructural white matter reorganization, we used diffusion tensor imaging and mapped the organization of large white matter bundles in CDKL5 KO males, Het females and sex-matched littermate controls. Voxelwise and regional assessments of fractional anisotropy (FA), a parameter sensitive to microscale white matter integrity [47], revealed the presence of largely preserved microstructure in all the major fiber tracts of KO and Het mice (CDKL5 KO: Fig. 1e, f, *q*  >  0.48, FDR-corrected; CDKL5 Het: Suppl. Fig. 2c, d, *q* > 0.54, FDR-corrected). The lack of regional FA differences argues against the presence of major alterations in whole-brain white matter topography, as these would be appreciable in the form of large regional FA differences [57]. To further evaluate microscale white matter integrity, we performed Myelin Basic Protein (MBP) staining of axonal fibers in the ACC [58]. In agreement with our FA data, we did not find differences in myelin thickness at the midline of the CC (Suppl. Fig. 3a, b) or the area covered by MBP within the ACC (Suppl. Fig. 3c) in adult CDKL5 KO mice, excluding severe interhemispheric axonal bundle alterations.

We next examined whether anatomical changes at the synaptic level would contribute to the FC increase. We performed immunohistochemistry for VGlut1+ and VGlut2+ excitatory presynaptic puncta across layers in the ACC of adult KO (Fig. 1g–i), labeling intracortical (VGlut1), callosal (VGlut1) and thalamocortical (VGlut2) excitatory synapses. We found a significant increase in the density of VGlut1+ puncta in adult CDKL5 KO (Fig. 1h, i, Suppl. Fig. 3d, e), without changes in VGlut2 (Suppl. Fig. 3d, f) suggesting a change in the number of cortico-cortical excitatory synapses. Altogether, these results argued against alterations of callosal axons, but rather pointed to abnormalities at the synaptic level and possible disruptions of synapse maturation.

### Increased long-range and decreased local excitatory transmission in CDKL5 mutant mice

**Fig. 2:**
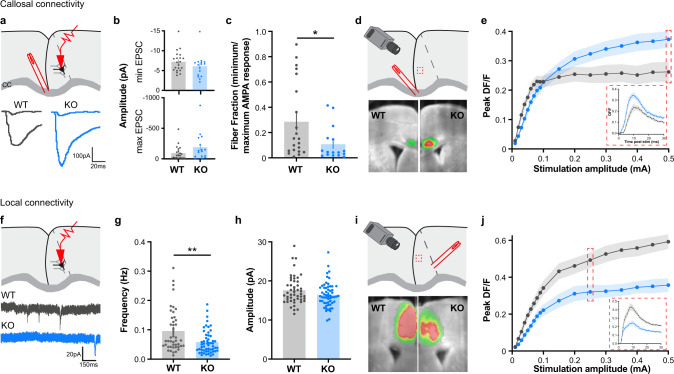
Differential alterations in long- and short-range excitatory inputs in the cingulate of juvenile CDKL5 KO mice. **a** (Top) Schematic of patch clamp recording configuration. (Bottom) Example traces of minimal and maximal eEPSP of L5 pyramidal neurons (Pyr) following electrical corpus callosum fiber stimulation. **b** Minimal eEPSC (top, WT: −7.211 ± 0.575 pA, *n* = 21 cells/3 mice, KO: −6.060 ± 0732 pA, *n* = 16 cells/3 mice, Mann-Whitney test, *p* = 0.1152) and maximal eEPSC (bottom, WT: −93.90 ± 25.19 pA, KO: −190.5 ± 55.68 pA, Mann-Whitney test, *p* = 0.1469) in cingulate pyramidal neurons. **c** Callosal fiber fraction at P15 (WT: 0.29 ± 0.07au, KO: 0.11 ± 0.03 au, Mann-Whitney test *p* = 0.0210*). **d** (Top) Schematic of voltage-sensitive dye imaging (VSDI) setting (Bottom) Example VSDI images of cingulate activity following electrical corpus callosum fiber stimulation. **e** Peak DF/F signal in layer 2/3 across increasing stimulation amplitudes of callosal fibers (Mixed-effects model REML, Interaction of factors: Genotype and Stimulation intensity, *p* < 0.0001***). (Insert) DF/F response over time at high stimulation (0.5 mA) (Two-way Anova, REML, interaction Genotype × Time, *p* = 0.045*; WT: n = 11slices/8mice, KO: n = 16slices/11mice). **f** (Top) Experimental schematic of patch clamp recordings (Bottom) Example traces of miniature EPSC (mEPSC) of L5 cingulate pyramidal neurons at P15. **g** Frequency of mEPSC (WT: 0.096 ± 0.011 Hz, *n* = 44 cells/4 mice; KO: 0.058 ± 0.005 Hz, *n* = 53 cells/4mice, Mann-Whitney test, *p* = 0.0028**). **h** mEPSC amplitude (WT: 17.61 ± 0.52pA, *n* = 48 cells/4mice; KO: 16.46 ± 0.42 pA, *n* = 57 cells/4 mice, Mann-Whitney test *p* = 0.1265). **i** (Top) VSDI configuration illustration. (Bottom) Example VSDI images of peak responses following layer 5/6 stimulation. **j** Peak DF/F responses in L2/3 following increasing stimulation (Mixed-effect model REML, interaction genotype × stimulation amplitude, *p* < 0.0001***, WT: *n* = 14 slices/6 mice, KO: *n* = 12slices/5mice). (Insert) DF/F response over time at high stimulation (0.25 mA) (Two-Way RM Anova, interaction genotype × stimulation amplitude, *p* < 0.0001***).

We reasoned that abnormalities found in adulthood may be the result of disrupted development of callosal synapses given that CDKL5 expression peaks in early postnatal development (~P15) and has been implicated in synapse maturation [35]. We therefore assessed functional synaptic connectivity of callosal projections in the ACC-RSP by obtaining the fiber fraction with whole-cell patch-clamp recordings of pyramidal neurons (Fig. 2a) at two stages of synapse development [49, 59, 60]. The number of callosal inputs innervating a cell is an inverse measure of the fiber fraction, the ratio of minimal and maximal AMPA responses of pyramidal neurons following CC stimulation [49, 61]. Thus, a low fiber fraction represents a large number of synapses, and vice versa. We found no differences at postnatal day 4 to 6 (P4–6) when synapses are forming (Suppl. Fig. 4a–c). Instead, there was a 70.6% decrease in the fiber fraction at P15-19 in CDKL5 KO mice compared to age-matched WT littermates, during refinement and maturation (Fig. 2a–c), indicating the presence of exuberant callosal synaptic inputs in juvenile mutants.

VSDI was then used to assess the impact on the overall activity across cortical layers. ACC-RSP cortical responses were measured in layer 2/3 (Fig. 2d, e) and layer 5/6 (Suppl. Fig. 4f–h) following CC stimulation in P15–19 to evaluate the input/output curve. We found peak responses (*ΔF*/*F*) in KO were significantly lower than WT at low stimulation, but significantly higher than WT at high stimulation. Activity from WT neurons reached a plateau around 0.1 mA, whereas the amplitude of the response continued increasing in KO mice, as reflected in the significantly different nonlinear regression fit of the two curves (*p* < 0.0001***, Fig. 2e). The response over time following stimulation at low amplitude (0.04 mA, Suppl. Fig. 4e) and high amplitude (0.5 mA, Fig. 2e insert) correlated with the differences observed at peak *ΔF*/*F*. The atypical responses were similar in deeper layers (Suppl. Fig. 4f–h) and correlate with an overall increase of the cortical area recruited over time following high stimulation (Suppl. Fig. 4i). Taken together, these results confirm the presence of weaker, but exuberant callosal inputs that may contribute to an enhanced activity in the ACC-RSP cortex of CDKL5 KO mice when strongly recruited.

Cortical pyramidal neurons receive most of their synaptic inputs from intrahemispheric neighboring neurons compared to callosal inputs [62]. We thus recorded miniature excitatory postsynaptic currents from cingulate neurons (mEPSCs, Fig. 2f). Interestingly, there was a significant reduction in the frequency, but not in the amplitude of mEPSCs in P15 CDKL5 KO mice (Fig. 2g, h). VSDI was then performed, and levels of activity were recorded in response to L5/6 intracortical stimulation (Fig. 2iL2/3; Suppl. Fig. 4k–l, L5/6). Recorded responses were significantly lower in KO mice compared to WT littermates at all tested stimulation intensities and across time (Fig. 2j; Suppl. Fig. 4k–l), and the curves of both genotypes were significantly different (nonlinear regression fit *p* < 0.0001***, Fig. 2j).

We then evaluated whether CDKL5 Het females share the same callosal and intracortical deficits. We found a significant decrease in the fiber fraction in P15–19 Het females compared to WT females (Suppl Fig. 2e–h), suggesting an increase in the number of callosal inputs. Additionally, we found a similar patterned response at the population level by VSDI following CC stimulation, where callosal responses were significantly stronger in Het at high stimulation in both upper and deeper layers (Suppl. Fig. 2i–k). On the other hand, local cingulate activity following L5/6 stimulation was moderately weaker in upper layers in Het females, although it was not as drastically disrupted as it was in the CDKL5 KO males (Suppl. Fig. 2l–n). Thus, CDKL5 Het females reliably recapitulate callosal impairments, suggesting callosal connectivity, unlike local circuitry, is particularly sensitive to gene dosage. Taken together, these results point at a differential effect of the loss of CDKL5 on intracortical (or local circuitry) vs. callosal excitatory connectivity in early postnatal development.

### Impaired cognitive function in adult CDKL5 KO mice

**Fig. 3:**
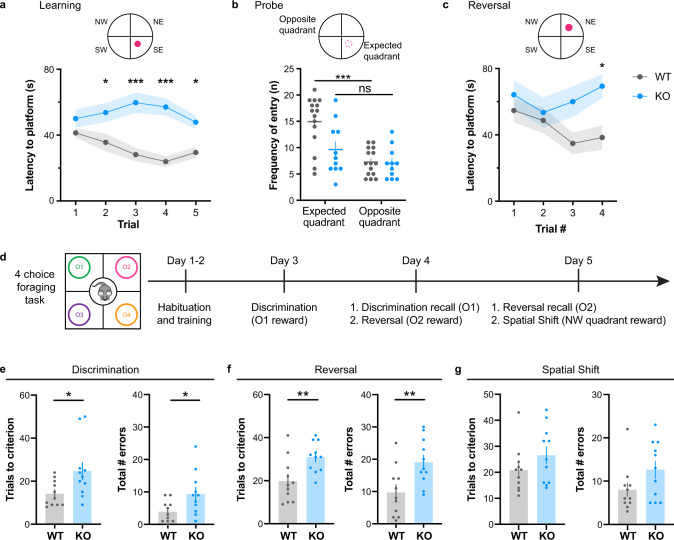
Cognitive impairment in CDKL5 KO mice. **a** Morris Water Maze learning paradigm (top) and latency to platform across 5 trials (two-way Anova, interaction trial x genotype *p* = 0.0429* , Sidak’s multiple comparison: trial 1: *p* = 0.6264, trial 2: *p* = 0.0280*, Trial 3: *p* < 0.0001***, trial 4: *p* < 0.0001***, trial 5: *p* = 0.0251* ). **b** Memory probe test where platform is removed (two way Anova, genotype *p* = 0.0174*, sidak’s multiple comparison between expected quadrant-opposite quadrant: WT: *p* < 0.0001***, KO: *p* = 0.2445). **c** Reversal task where platform is moved to another quadrant (top) and latency to platform is measured across four trials (two-way Anova, genotype *p* = 0.0021**, Sidak’s multiple comparison, Trial 1: *p* = 0.8669, Trial 2: *p* = 0.9880, Trial 3: *p* = 0.0998, Trial 4: *p* = 0.0261*) (WT: 15 mice, KO: 11 mice). **d** Schematic description of four-choice foraging task paradigm, where O1 is rewarded during the discrimination phase (day3), O2 is rewarded during the reversal (day 4), and NW quadrant rewarded during the spatial shift task (day 5). **e** (Left) Number of trials required to reach criterion during the initial discrimination task (consecutive 8/10 correct trials; WT: 14.18 ± 1.78, KO: 24.73 ± 4.01, Mann Whitney *p* = 0.0196*). (Right) Total number of errors made show an impairment in the KO mice (WT: 3.91 ± 0.99, KO: 9.37 ± 2.01, unpaired t test *p* = 0.0241*). **f** (Left) Number of trials to reach criterion during the reversal task (WT: 19.82 ± 3.06, KO: 30.91 ± 2.10, Unpaired t-test *p* = 0.0073**). (Right) Number of errors made during the reversal task (WT: 9.73 ± 2.33, KO: 19.09 ± 2.16, unpaired t-test *p* = 0.0080**). **g** (Left) Number of trials to reach criterion in the spatial shift (WT: 20.91 ± 2.66, KO: 26.55 ± 3.28, unpaired t test *p* = 0.1965). (Right) Total number of errors during the task (WT: 8.00 ± 1.58, KO: 12.64 ± 1.98, unpaired t-test *p* = 0.1103). Mean ± SEM.

To test the impact of miswired prefrontal circuits on cognition, we performed the classical Morris Water Maze (MWM) test plus an odor-guided four-choice foraging task involving rule learning, decision-making, and exploitation/exploration behaviors. The ability to conduct such complex tasks requires the proper maturation of PFC circuits, including the ACC-RSP [51, 63]. In the MWM, CDKL5 KO mice took significantly longer to learn the location of the hidden platform in a 5-trial learning phase (Fig. 3a) as previously reported in CDKL5 exon 4 germline deletion mouse model [52, 64]. Memory was evaluated in the probe trial, where the platform was removed. Unlike WT mice, KO mice entered the expected quadrant and the opposite quadrant at equal frequency, suggesting memory impairment (Fig. 3b). On a reversal task, KO mice took longer to learn the new location of the platform (Fig. 3c), showing a deficit in cognitive flexibility. We then evaluated the mice in a four-choice foraging test (Fig. 3d) where they must find a food reward hidden in a bowl of wood shavings masked by four different odors. After habituation and training, the mice learn the first association of an odor (anise) to the food reward in the discrimination task, which changes location after each 3 min trial. This association was then re-affirmed the next day, before proceeding to a reversal task, where a new odor (clove) masked the reward. The final day, this new association was tested in a recall task, and then proceeded to a paradigm shift, where a specific location (NW), and not an odor was hiding the reward. In the initial discrimination phase, adult CDKL5 KO mice required significantly more trials to reach criterion (8/10 consecutive trials) and made significantly more errors than WT mice (Fig. 3e). Nevertheless, CDKL5 KO mice were able to remember the food-odor association the following day during a recall task (Suppl. Fig. 5a). Mice then underwent a reversal task where CDKL5 KO mice also required significantly more trials to reach criterion and made significantly more errors than WT (Fig. 3f). Notably, KO mice made more reversal errors by choosing the bowl with the previously rewarded odor (anise), or the novel unrewarded odor that was introduced in this task (eucalyptus) indicating both impairment in cognitive flexibility, and curiosity/odor preference (Suppl. Fig. 5c). Eventually, KO mice learnt the new task (Suppl. Figure 5b). In the final spatial shift task, there was a non-statistically significant trend for CDKL5 KO to make more errors than WT (Fig. 3g, *p* = 0.11). Note that the differences were not due to inability to detect odors as we did not find any impairment in the odor habituation/dishabituation test (Suppl. Fig. 5e). Taken together, our results indicate a significant impairment in PFC-dependent cognitive function and flexibility in the absence of CDKL5.

### Loss of CDKL5 in CPN enhances callosal synaptic connectivity

**Fig. 4:**
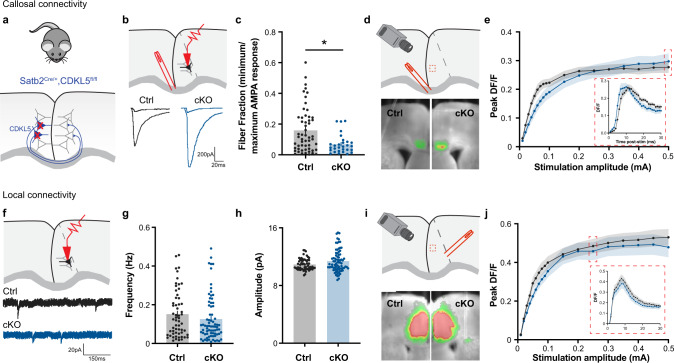
Selective deletion of CDKL5 from Satb2+ callosal projection neurons display similar callosal deficits. **a** Schematic of breeding cross of Satb2^cre/+^ and CDKL5^fl/fl^ mouse lines. **b** (Top) Patch clamp recordings schematic of P15 L5 pyramidal neurons in the cingulate cortex following corpus callosum stimulation. (Bottom) Example traces of minimal and maximal eEPSP responses. **c** Fiber fraction at P15 demonstrating an increase in the number of callosal inputs in conditional KO (cKO) mice (Mann Whitney *p* = 0.0072**, Ctrl: 0.158 ± 0.019, *n* = 56 cells/8 mice; cKO: 0.069 ± 0.011, *n* = 31 cells/6 mice). **d** (Top) Schematic of VSDI configuration (Bottom) Example VSDI images of cingulate activity following electrical corpus callosum fiber stimulation. **e** Peak DF/F signal in layer 2/3 across increasing stimulation amplitudes of callosal fibers (Two-Way Anova REML, Interaction of factors: Genotype and Stimulation intensity, *p* = 0.7519). (Insert) D*F*/*F* response over time at high stimulation (0.5 mA) (Two-way Anova, interaction Genotype × Time, *p* < 0.0001***; Ctrl: *n* = 11 slices/9 mice, KO: *n* = 12 slices/9 mice). **f** Patch clamp schematic recordings of layer 5 cingulate pyramidal neurons from P15 (Top) with example traces of mEPSC (Bottom). **g** Frequency of mEPSC (Mann Whitney *p* = 0.2058, Ctrl: 0.151 ± 0.016, *n* = 59 cells/3 mice; cKO: 0.127 ± 0.013, *n* = 75 cells/3 mice). **h** mEPSC amplitude (Mann Whitney *p* = 0.1608, Ctrl: 10.92 ± 0.104, *n* = 59 cells/3 mice, cKO: 11.39 ± 0.172, *n* = 79 cells/3 mice). **i** (Top) VSDI configuration illustration. (Bottom) Example VSDI images of peak responses following local layer 5/6 fiber stimulation. **j** Peak DF/F responses in L2/3 following increasing stimulation intensity (Two-Way Anova, REML, interaction genotype × stimulation amplitude, *p* = 0.9912, Ctrl: *n* = 12 slices/6 mice, cKO: *n* = 11 slices/6 mice). (Insert) D*F*/*F* response over time at high stimulation (0.25 mA) (Two-Way Anova, interaction genotype × time, *p* = 0.7429).

To test the hypothesis that the loss of CDKL5 only in CPN is necessary for proper callosal functional connectivity, we selectively removed CDKL5 from Satb2-positive neurons by crossing CDKL5^fl/fl^ mice with Satb2^cre/+^ animals (Fig. 4a). Satb2 is expressed in the predominant excitatory CPN subtype and is required for interhemispheric axonal projections [65]. The number of callosal inputs was first evaluated by measuring the fiber fraction of P15-19 neurons in Satb2^cre/+^,CDKL5^fl/y^ conditional mice (termed cKO from hereon) compared to control littermates (Satb2^+/+^, CDKL5^fl/y^ and Satb2^cre/+^,Cdkl5^+/y^ - termed Ctrl from hereon). Similarly to CDKL5 KO animals, cKO mice exhibited a significantly smaller fiber fraction at P15 compared to Ctrl mice (Fig. 4b, c, Suppl. Fig. 6a, b), suggesting an increased number of callosal inputs. We then recorded cortical network activity following CC stimulation by VSDI (Fig. 4d). Although the overall level of activity did not increase as much in cKO mice as in KO mice, the response curve following increasing stimulation strength was comparable to what was observed in the KO cingulate cortex (Fig. 4e). Particularly, at low stimulation, CDKL5 cKO mice exhibited a lower *ΔF*/*F* response than Ctrl, and while the slope plateaus in the Ctrl around 1.5 mA, the slope in the cKO mice progressively increases as the stimulation intensity rises, paralleling KO mice responses (Fig. 4e; nonlinear regression *p* = 0.0008***). There was also a shift in the timing of the response at high intensity stimulation, where the cKO responses were faster compared to Ctrl, suggesting a possible change in the location of callosal inputs onto cingulate neurons (Fig. 4e insert, Suppl. Fig. 6c). Similar deficits were found in layer 5/6 (Suppl. Fig. 6e–g) supporting an increase in the number of callosal inputs, albeit less severe than in the constitutive KO mice.

To evaluate changes in local circuits, we then recorded mEPSCs in P15 cKO mice (Fig. 4f). In contrast to KO mice, we did not detect a significant difference either in the frequency (*p* = 0.21, Fig. 4g) or amplitude (*p* = 0.16, Fig. 4h) between cKO and Ctrl cingulate pyramidal neurons. In addition, VSDI recordings in response to intracortical L5/6 stimulation (Fig. 4i) did not reveal any significant differences in the *ΔF*/*F* responses across stimulation amplitude (Fig. 4j), pointing to a selective impairment in callosal excitatory circuitry.

### Selective impairment of  CPN connectivity is sufficient to disrupt cognitive function

**Fig. 5:**
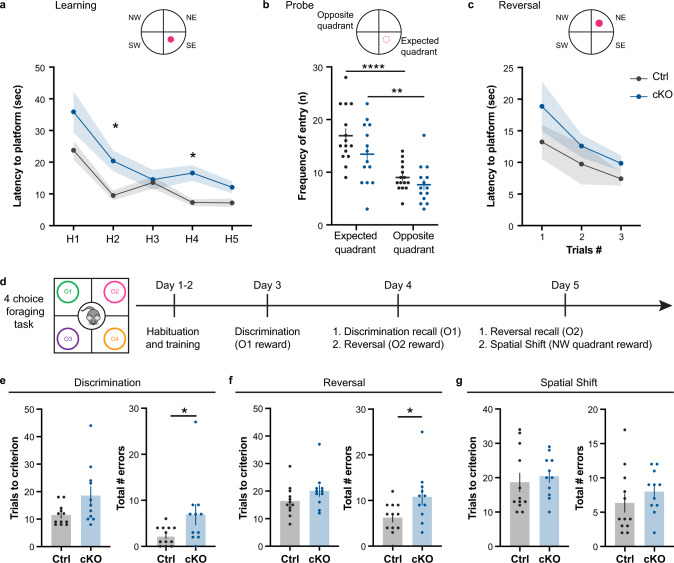
Cognitive impairment in Satb2 conditional KO. **a** Latency to platform during the 5 trials in learning phase in control and cKO mice (two-way Anova, interaction trial x genotype, *p* = 0.1865, Sidak’s multiple comparison: trial 1: *p* = 0.4394, Trial 2: *p* = 0.0334*, Trial 3, *p* = 0.9998, Trial 4: *p* = 0.0143*, Trial 5:0.1991). **b** Memory was then tested the following day (two-way Anova, Sidak’s multiple comparison within genotypes between expected vs. opposite quadrant, Ctrl: *p* < 0.0001, cKO: *p* = 0.0024). **c** Latency to platform when it has moved to a new location (two-way Anova, interaction genotype × trial, *p* = 0.6529, Sidak’s multiple comparison, Trial 1: *p* = 0.5732, Trial 2, *p* = 0.8332, Trial 3: 0.4021). Ctrl: *n* = 15 mice, cKO: *n* = 14 mice. **d** Four-choice foraging task paradigm. **e** (Left) Number of trials needed to reach criterion during the discrimination task (Ctrl: 11.50 ± 1.06, *n* = 12mice; cKO: 18.55±3.26, *n* = 11mice, Mann-Whitney test *p* = 0.065). (Right) Total number of errors during the discrimination task (Ctrl: 2.33 ± 0.60, cKO: 6.91 ± 2.21; Mann-Whitney test *p* = 0.0439*). **f** (Left) Number of trials to reach criterion during the reversal phase (Ctrl: 16.42 ± 1.549; cKO: 20.09±1.979, Mann-Whitney test *p* = 0.1132). (Right) Total number of errors during the reversal task (Ctrl: 6.250 ± 0.962, cKO: 10.82 ± 1.742, unpaired t-test *p* = 0.0287). **g** (Left) Number of trials to reach criterion in the spatial shift (Ctrl: 18.67 ± 2.647; cKO: 20.45 ± 1.826, Mann-Whitney test *p* = 0.3693). (Right) Total number of errors during the task (Ctrl: 6.333 ± 1.345, cKO: 8.00 ± 0.9439, Mann-Whitney test *p* = 0.1926). Mean ± SEM.

We assessed cognitive performance in cKO and Ctrl mice. In the MWM, cKO took significantly longer than their littermate controls to find the hidden platform (Fig. 5a). However, they did not present any spatial memory deficit tested in the probe trial (Fig. 5b) or flexibility deficits in the reversal task (Fig. 5c). In the foraging task (Fig. 5d), cKO mice, like KO animals, made significantly more errors during discrimination (Fig. 5e), and reversal (Fig. 5f). The cKO mice did not show deficits during the recalls, suggesting no significant impairment in memory formation (Suppl. Fig. 7a, b), nor demonstrated deficits in the spatial shift paradigm (Fig. 5g). The olfactory habituation/dishabituation test also demonstrated no olfactory deficits in cKO (Suppl. Fig. 7e). Overall, our results supported the working hypothesis that CDKL5 in CPN is necessary to allow proper functional callosal connectivity and cognitive flexibility.

### Selective CDKL5 expression in Satb2 + CPN prevents cognitive impairment

**Fig. 6:**
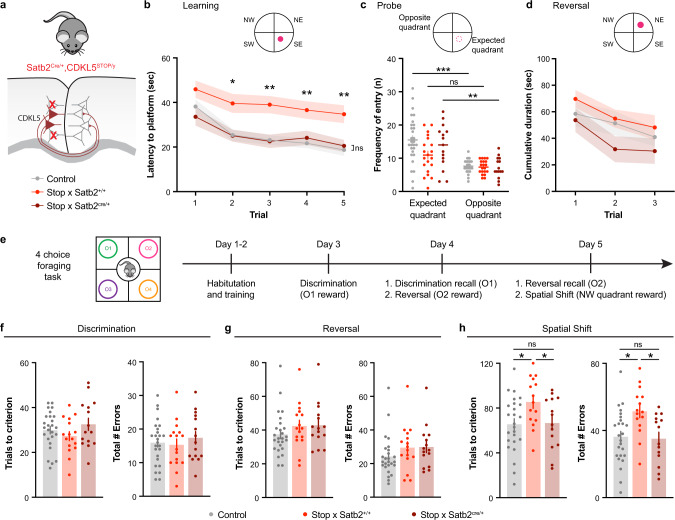
Cognitive impairment rescued in CDKL5 specific expression in Satb2 + CPN. **a** Latency to platform during the five trials in the learning phase. CDKL5^STOP^ × Satb2^cre/+^ mice learn at the same pace as controls, and both of these groups are significantly different to CDKL5^STOP^ × Satb2^+/+^ deficient mice (two-way Anova, genotype *p* = 0.0002***, significant trial 2–5^#^). **b** Probe test reveals memory impairment in Stop × Satb2^+/+^ mice (two-way Anova, Sidak’s multiple comparison between opposite vs expected quadrant: Ctrl *p* < 0.0001***, Stop × Satb2^+/+^ *p* = 0.0667, Stop x Satb2^cre/+^ *p* = 0.0022**). Ctrl: *n* = 29 mice (Satb2 cre- and Satb2 cre+ CDKL5 WT controls combined), StopxSatb2^+/+^: *n* = 20 mice, Stop × Satb2^cre/+^: *n* = 15 mice. **c** Latency to platform in reversal test (two-way Anova, interaction genotype × trial, *p* = 0.912). Ctrl: *n* = 13 mice, StopxSatb2^+/+^: *n* = 13 mice, Stop × Satb2^cre/+^: *n* = 8 mice. **d** Four-choice foraging task paradigm. **e** (Left) Number of trials needed to reach criterion during the discrimination task (Ctrl: 29.9 ± 1.6, StopxSatb2^+/+^: 27.3 ± 2.0, Stop × Satb2^cre/+^: 32.5 ± 2.7, One-way Anova, *p* = 0.2596). (Right) Total number of errors during the discrimination task (Ctrl: 16.0 ± 1.3, StopxSatb2^+/+^: 15.3 ± 1.7, Stop x Satb2^cre/+^: 17.40 ± 1.8, One-way Anova, *p* = 0.6869). **f** (Left) Number of trials to reach criterion during the reversal phase (Ctrl: 37.5 ± 2.5, StopxSatb2^+/+^: 42.4 ± 3.4, Stop × Satb2^cre/+^: 42.8 ± 3.4, One-way Anova, *p* = 0.3475). (Right) Total number of errors during the reversal task (Ctrl: 24.2 ± 2.4, StopxSatb2^+/+^: 29.5 ± 3.4, Stop × Satb2^cre/+^: 29.9 ± 3.3, One-way Anova, *p* = 0.2715). **g** (Left) Number of trials to reach criterion in the spatial shift (Ctrl: 65.5 ± 4.9, Stop × Satb2^+/+^: 85.4 ± 5.7, Stop × Satb2^cre/+^: 66.6 ± 6.3, One-way Anova, *p* = 0.0311*). (Right) Total number of errors during the task (Ctrl: 36.2 ± 3.2, StopxSatb2^+/+^: 51.4 ± 3.7, Stop × Satb2^cre/+^: 35.1 ± 3.9, One-way Anova, *p* = 0.0059**). Ctrl: *n* = 26 mice, StopxSatb2^+/+^: *n* = 16mice, Stop × Satb2^cre/+^: *n* = 15mice. ^#^See supplementary table for detailed statistics. Mean ± SEM.

We next sought to determine whether expression of CDKL5 solely in Satb2+ CPN was sufficient to rescue the cognitive deficit observed in CDKL5-deficient mice. We used CDKL5^STOP^ mice, which carry a lox-P-flanked transcriptional STOP cassette in the exon 3 of endogenous CDKL5 gene [42], lack functional CDKL5 protein and recapitulate many phenotypes observed in the exon 6 CDKL5 KO [37, 42]. They were crossed with Satb2^Cre/+^ mice to allow CDKL5 expression selectively in Satb2+ neurons (Fig. 6a). Learning and memory were first tested through the MWM test. Consistent with the results in CDKL5 KO mice, Satb2^+/+^, CDKL5^Stop/y^ mice (hereon Stop × Satb2^+/+^) required more time to learn the location of the hidden platform compared to control littermates (Fig. 6b). Interestingly, Satb2^cre/+^, CDKL5^Stop/y^ mice (referred to as Stop × Satb2^cre/+^, with CDKL5 expressed in Satb2+ cells) exhibited a learning behavior overlapping that of their control littermates and significantly different from Stop × Satb2^+/+^ mice. During the probe test, Stop × Satb2^+/+^ mice exhibited a deficit in spatial memory as the frequency of entry was not different between the expected and opposite quadrant (Fig. 6c) unlike their control littermates, and the Stop × Satb2^Cre/+^ rescue animals. We found no statistical difference between any groups during the reversal task (Fig. 6d). We then performed the mPFC-dependent four-choice foraging task (Fig. 6e). Although, we did not find any significant difference between any groups in the discrimination (Fig. 6f) and reversal tasks (Fig. 6g), the Stop × Satb2^+/+^ mice exhibited a significant increase in the number of trials needed to reach criterion and number of errors in the more complex spatial rule shift paradigm (Fig. 6h, Suppl. Fig. 8a–d). Most notably, the deficits that were observed in these CDKL5-deficient mice were absent in the Stop × Satb2+ re-expression group, which behaved like their control littermates. We confirmed that they did not have any olfactory deficits (Suppl. Fig. 8e). These results further support the hypothesis of a direct involvement of interhemispheric connectivity in cognitive performance in CDKL5-deficient mice.

## Discussion

This study reveals a novel role of CDKL5 by demonstrating that it is both necessary and sufficient for proper CPN connectivity and cognitive flexibility, and it also supports a causal relationship between CPN dysfunction and cognitive impairment in CDD and potentially related NDD.

In the absence of CDKL5, we found a significant increase in the number of callosal inputs and corresponding cingulate activity, suggesting its involvement in callosal synaptic maturation. This synaptic exuberance may result from either an increase in synapse formation, or a delay/failure in synapse refinement. Between P5 and P15, the fiber fraction of WT animals increased (Fig. 2c, Suppl. Fig. 3c [49]), suggesting a decrease in the number of callosal inputs and thus, successful pruning of weak synapses. In addition, the density of excitatory presynaptic markers VGlut1 and VGlut2 increased until P30, and decreased by adulthood in WT animals, further supporting a morphological pruning of excitatory synapses (Suppl. Fig. 2c–d). On the contrary, the fiber fraction decreased and VGlut1+ synaptic density remained high from P15 onwards in the KO mice, suggesting there may be both an increase in synapse formation and a failure of refinement of callosal neurons resulting in an overall deficit in synapse development. Further dissection of callosal vs. local VGlut1+ puncta is needed to confirm whether such increase is specific to callosal synapses. Finally, while CDKL5 regulates postsynaptic stability and dendritic development [35], our results suggest it may also play a role presynaptically. Whether this role is indirect through CDKL5’s contribution to microtubule dynamics [66, 67], or direct through the targeting of vesicle release or by interfering with an elimination signal in weak synapses remains to be explored.

Our findings suggest there is a reorganization of cortico-cortical inputs, with CDKL5 differentially impacting excitatory neuron subtypes. A possibility for the imbalance of excitatory inputs may result as a compensatory mechanism, such that the increase of callosal inputs drives the reduction of local connectivity, or vice-versa. However, with mosaic CDKL5 expression in Het females, the callosal impairments were still present, while the intracortical deficits were not as severe as in the complete absence of CDKL5. Similarly, Satb2^cre/+^, CDKL5^fl/y^ conditional KO mice showed an increase of callosal inputs with no effect on local excitatory connectivity. These results raise the possibility that CDKL5 has distinct cell autonomous roles in short- vs. long-range excitatory projection neurons and suggests that callosal neurons are particularly sensitive to the levels of CDKL5. The underlying molecular mechanisms still need to be explored. CPN may also disproportionately target inhibitory interneurons that in turn may silence excitatory local networks. In fact, CPN innervate parvalbumin (PV) + GABAergic interneurons directly and contribute to feedforward inhibition to suppress L5 cortico-cortical pyramidal neurons [68]. Interestingly, there is a specific increase in PV + interneurons in the visual cortex of CDKL5 KO mice starting from P35 and an increase in VGlut1+ puncta contacting PV + dendrites [69]. Thus, we cannot exclude the possibility that the increase in VGLUT1 + puncta found in adulthood target PV + interneurons. Moreover, there is a small population of GABAergic CPN which have been identified in sensory and motor cortices [70]. Future studies should elucidate how GABAergic CPN and local inhibitory neurons contribute to interhemispheric connectivity and to higher order cognitive function in the absence of CDKL5.

At the network level, cingulate neurons receive convergent inputs from callosal, local cortico-cortical afferents and thalamocortical projections that together contribute to the expression of complex behaviors. Interestingly, in early development, layer 4 excitatory neurons in primary somatosensory and visual cortices extend transient callosal projections before only projecting ipsilaterally. Perturbations of thalamic inputs result in the persistence of such ectopic callosal afferents, their functional maturation and integration in the overall circuit [71]. While we did not find a significant increase in VGlut2+ density, possible thalamocortical synaptic impairments may be present and contribute to circuit deficits. Additionally, recent findings suggest that inhibition of mPFC increases rs-fMRI connectivity in PFC and its thalamo-cortical targets [72]. It is thus possible that a disruption in long-range thalamocortical inputs, as a direct or indirect effect of the loss of CDKL5, may also contribute to the functional and behavioral deficits, particularly as the thalamus participate in controlling cortical connectivity and synchronization across multiple regions to maintain rule representation [73–75].

The significance of changes in long- vs. short-range functional connectivity has been a central focus in NDD research. Here, we found that interhemispheric long-range connectivity promotes proper cognitive performance. Although we cannot exclude that the decrease in local connectivity may also contribute to the cognitive impairments of CDKL5 KO mice, the behavioral deficits were largely recapitulated in Satb2+ conditional KOs and rescued in mice with Satb2-specific CDKL5 expression. These results strongly support the idea that disrupted callosal interhemispheric connectivity drives cognitive deficiencies in CDD. A limitation of the employed genetic approach is that *Satb2* is expressed in CPN across cortices and in a subset of CA1 hippocampal neurons possibly contributing to the spatial learning deficits/rescue we observed in the MWM. However, the reversal task of the MWM and the foraging task are modulated by the PFC/ACC networks, which motivated the use of such behavioral paradigms. Moreover, while CDKL5 KO mice have demonstrated increased susceptibility to seizure generation [39, 76], the absence of early-life spontaneous and recurrent seizures in this animal model allowed us to identify the consequence of CPN dysfunction independently from severe spontaneous seizures; suggesting that seizures alone are not responsible for the developmental perturbations and cognitive impairment described in CDD patients.

It is increasingly clear that neural connectivity miswiring represents a core endophenotype of NDD. Although the directionality of changes cannot be generalized in part due to the heterogeneity of NDD, common motifs emerge between clinically similar disorders. When functional connectivity of 16 animal models of ASD-risk genes were evaluated, CDKL5- deficient mice clustered with *Mecp2*-deficient mice, a model for Rett Syndrome, whose patients share many common phenotypes with CDD patients [77]. Considering our results, it would be interesting to analyze whether interhemispheric connectivity disruption is similar within clusters, or whether it could be used to further refine stratification. Evaluating interhemispheric structural and functional connectivity should also be considered in patients to evaluate whether the deficits observed here translate to humans. Finally, our findings may pave the way for more targeted pharmacological treatment, given the cell subtype specific changes in the absence of CDKL5; and support therapeutic interventions that restore CDKL5 expression in a pathway-specific and dose-dependent manner such as protein substitution therapy [52] or gene therapy [78, 79] shown to be beneficial in mice.

## Supplementary information

Supplementary Figures

Supplementary material and methods

Reporting summary

Supplementary Statistics information
