# Supplementary material for: CDKL5 sculpts functional callosal connectivity to promote cognitive flexibility
Source: Mol Psychiatry. 2023 Feb 3;29(6):1698–709. doi: 10.1038/s41380-023-01962-y (PMC11371650; doi:10.1038/s41380-023-01962-y)
Supplement: Supplementary file 1 — Supplementary Figures [file 41380_2023_1962_MOESM1_ESM.pdf]

Supplementary Figure 1

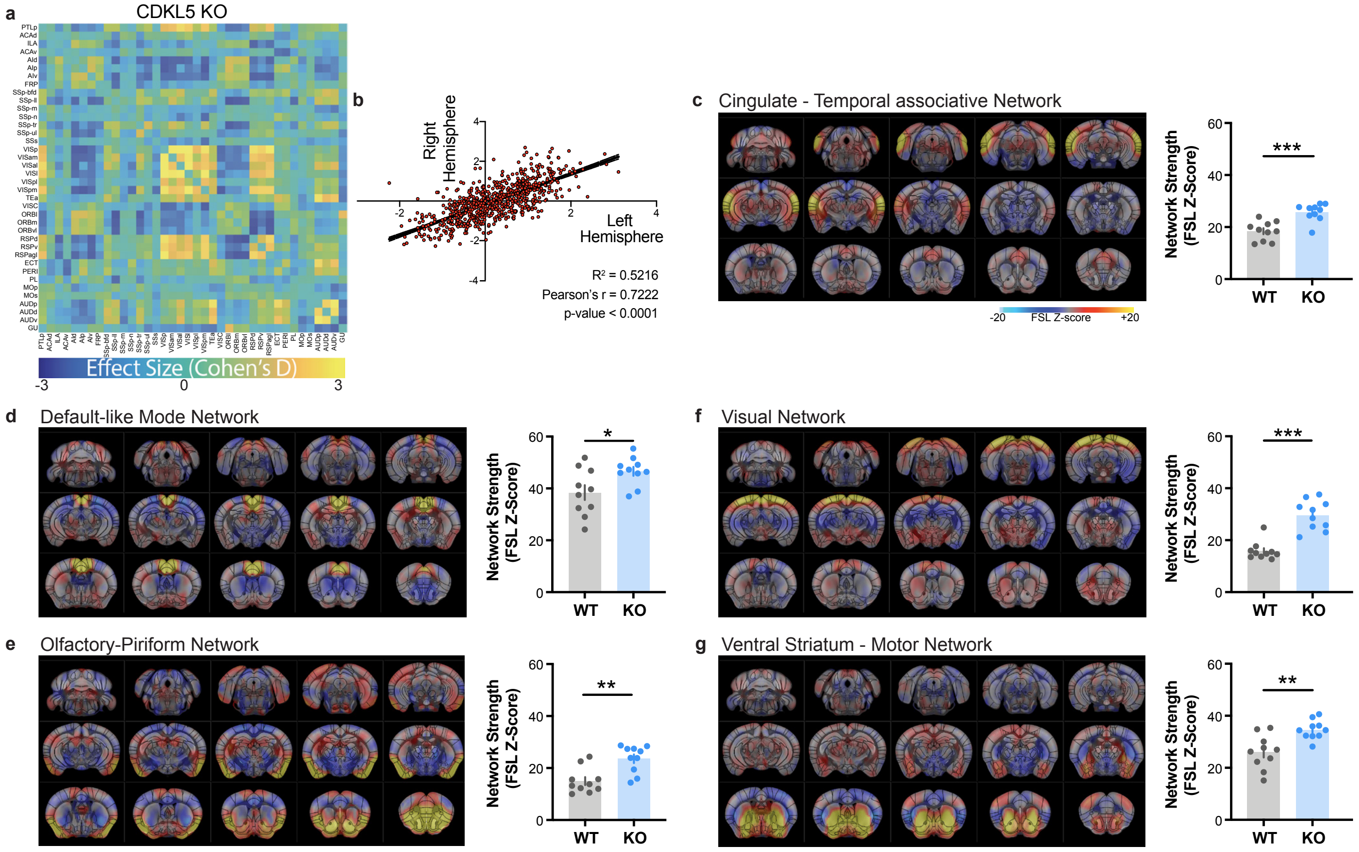

Supplementary Figure 1. Functional connectivity alterations in cortical and sensory networks in adult CDKL5 KO mice. a. Matrix showing the effect size between CDKL5 WT and KO across all regions. These are the edges-data from which node-date are thereafter calculated in figure1b. **b**. Associated graph showing agreement between connectivity deficits found in the right and left hemispheres. **c**-**g**. (Left) 2D anatomical representation of hyper-connectivity (red) or hypo-connectivity (blue) in areas involved in known networks. **c**-**g**. (Right) Independent component analysis (ICA)-based network analysis reveals hypersynchronization in cingulate-Temporal associative network (**c,** WT: 18.44±1.152, KO: 25.80±1.061, unpaired t-test p=0.0002***) and the default-like mode network (**d**, WT: 38.43±2.841, KO: 46.61±1.728, unpaired t-test p=0.0242*). Sensory-related areas also demonstrated hyperconnectivity (**e**, olfactory-piriform network: WT: 15.01±1.539, KO: 23.60±1.659, unpaired t-test p=0.0013**; **f**, visual network (WT: 15.84±1.100, KO: 29.64±1.810, Mann-Whitney p<0.0001***; **g**, ventral striatum and motor network (WT: 26.06±2.060, KO: 34.63±1.163, unpaired t-test p=0.0020**) (n=10/group). Mean±SEM.

Supplementary Figure 2

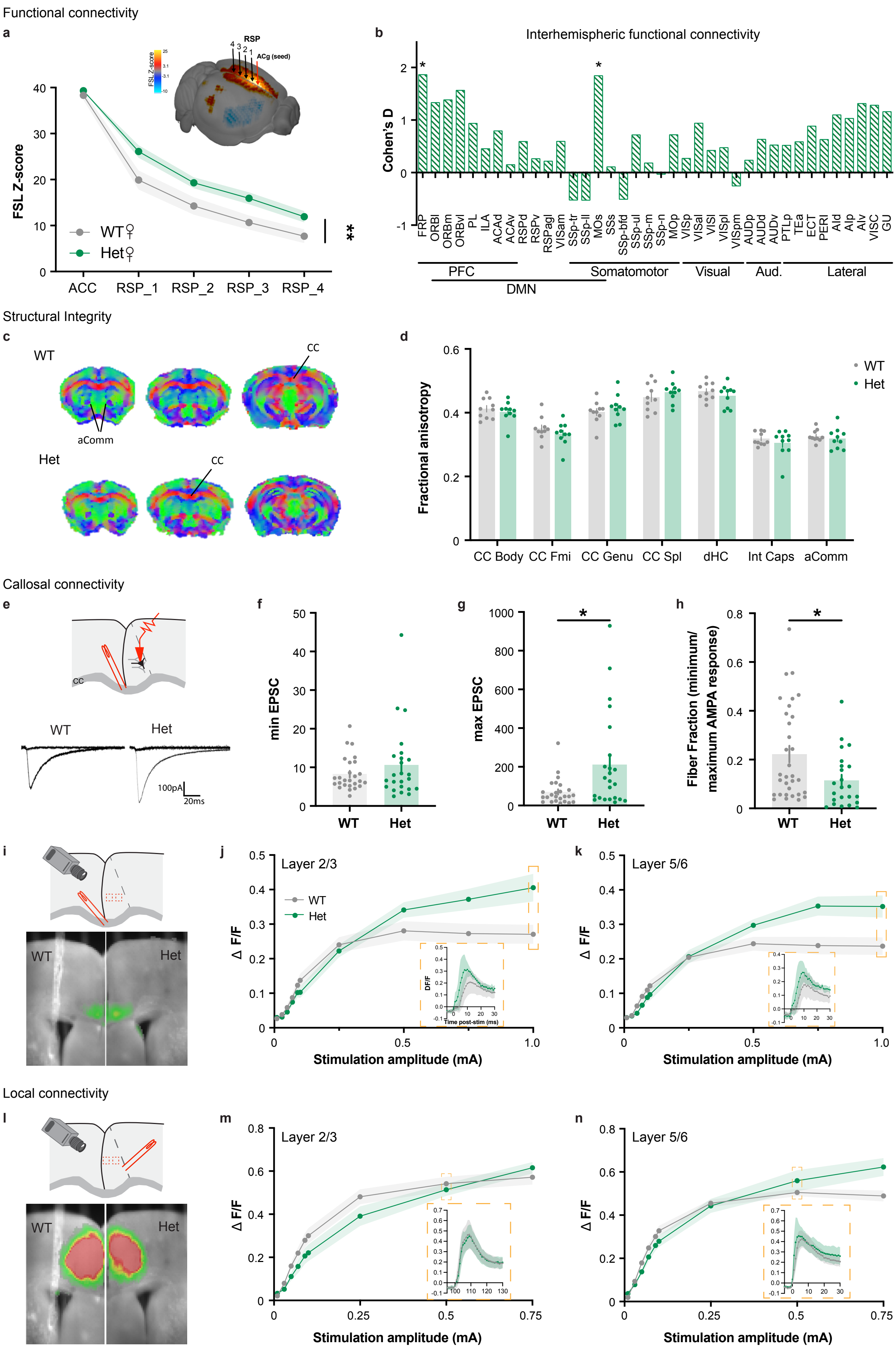

Supplementary Figure 2. Callosal deficits in ACC-RSP connectivity in CDKL5 Het females. a. Connectivity coupling of seed-based analysis originating from the ACC was measured using Z-scored normalized Pearson's correlation coefficient (FSLNets; two-way Anova, region $F_{2.807, 50.52}$ = 492.4, p<0.0001, genotype: $F_{1,18}$ = 5.722, p=0.0279*, Region x Genotype $F_{4,72}$=3.835, p=0.0070**). **b**. Cohen D's effect size of interhemispheric functional connectivity evaluated in different brain regions between WT and Het female mice (n=10 WT and 11 Het females, multiple t-test, Bonferroni corrected). **c**. Fractional anisotropy (FA) brain maps in a representative WT (top) and Het (bottom) mouse. Images are color coded by the first eigenvector of the FA, red for the left-right, green for anterior-posterior and blue for top-bottom direction. **d**. Quantification of FA in the anterior commissure, corpus callosum, dorsal hippocampus, and internal capsule as assessed with diffusion-weighted imaging. (multiple t-test FDR corrected, p>0.05 all regions). CC Body, body of the corpus callosum; CC Fmi, forcep minor of the corpus callosum; CC Genu, genu of the corpus callosum; CC Spl, splenium of the corpus callosum. n = 10 mice/group. **e.** (Top) Experimental schematic (Bottom) Example traces of minimal and maximal eEPSP responses. **f**. ACC neurons minimal eEPSC (WT: 8.27±0.85pA, n=26cells/3mice, Het: 10.65±1.92pA, n=24cells/4mice, Mann-Whitney test, p=0.8099) **g**. Maximal eEPSC in cingulate pyramidal neurons (WT: 70.50±13.51pA, Het: 212.2±51.35pA, Mann-Whitney test, p=0.0293*). **h**. Callosal fiber fraction at P15 (WT: 0.22±0.19au, Het: 0.11±0.23au, Mann-Whitney test p=0.0181*). **i**. (Top) VSDI experimental schematic (Bottom) Example images of cingulate activity following electrical corpus callosum fiber stimulation. **j-k**. Peak ΔF/F signal in layer 2/3 (**j**) and layer 5/6 (**k**) across increasing stimulation amplitudes of callosal fibers (Two-Way Anova REML, Interaction of factors: Genotype and Stimulation intensity p<0.0001*** for both L2/3 and layer 5/6, WT: n= 18slices/8mice, KO: n= 14slices/6mice). (Insert) ΔF/F response over time at high stimulation (1mA) (**j**, layer 2/3, Two-way RM Anova, interaction Genotype x Time, p<0.0001***; **k**, layer 5/6 Two-way Anova, interaction Genotype x Time, p<0.0001***). **l**. (Top) VSDI configuration illustration. (Bottom) Example VSDI images of peak responses following local layer 5/6 fiber stimulation. **m-n**. Peak ΔF/F responses in L2/3 (**m**) and layer 5/6 (**n**) following increasing stimulation intensity (**m**, Two-Way Anova, REML, interaction genotype x stimulation amplitude p=0.0061**; **n**, Two-Way Anova, REML, interaction genotype x stimulation amplitude p<0.0001***, WT: n=19slices/9mice, Het: n= 13slices/7mice). (Insert) ΔF/F response over time at high stimulation (0.25mA) (**m**, L2/3, Two-Way RM Anova, interaction genotype x time p>0.9999); **n**, L5/6, Two-Way Anova, interaction genotype x time p<0.0001***). Mean±SEM.

Supplementary Figure 3

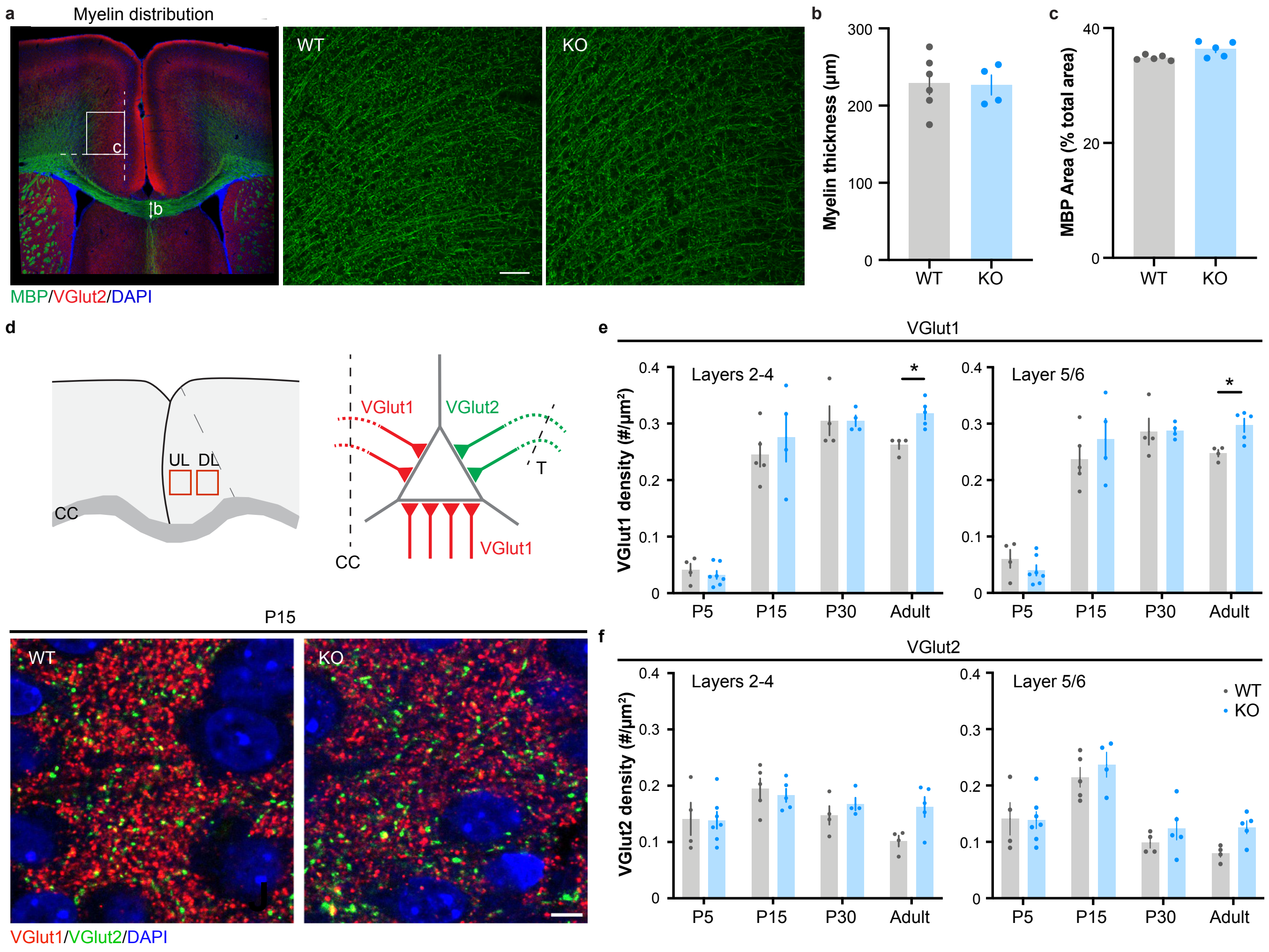

Supplementary Figure 3. Developmental anatomical alterations in CDKL5 KO mice. a. (Left) Anatomical evaluation of the axonal tract of the corpus callosum by staining of Myelin Basic Protein (MBP). (Right). Representative image of adult MBP staining in the deeper layers of cingulate cortex of CDKL5 WT and KO mice (Scale bar, 50µm). **b**. Myelin thickness measured at the representative line at the midline of the corpus callosum in **c**. (WT: 229.0±14.7 n= 6mice (35 sections), KO: 226.7±12.9, n= 4mice (22 sections), Mann Whitney test, p=0.9143). **c**. Measure of the area covered by MBP in the delineated square in **a**. The area analyzed covers the deeper layers of the cingulate cortex, and is delineated by L4 staining of Vglut2, and the peak of corpus callosal in cortex (WT: 34.83±0.21, n= 5mice (28 section); KO: 36.34±0.60, n= 5 mice (27 sections), Mann-Whitney, p=0.0952). **d**. (Top) Schematic of region of interest in the cingulate cortex, in upper layers 2-4 and deeper layers 5-6 and schematic representation of VGlut1 staining labeling both callosal and intracortical synapses (red), while VGlut2 labels excitatory thalamo-cortical synapses (green, CC: Corpus Callosum, T: Thalamus). (Bottom) Example images of glutamatergic transporter markers VGlut1 (red) and VGlut 2 (green) and DAPI (blue) staining in P15 samples. Scale bar, 5µm. **e**. VGlut1 density across development in layers 2 to 4 (left, multiple t-test, Holm-Sidak multiple comparison correction, P5: p=0.865, P15: p=0.865, P30: p>0.99, adult: p=0.0197*) and layer 5/6 (right, multiple t-test, Holm-Sidak multiple comparison correction, P5: p=0.587, P15: p=0.662, P30: p=0.952, adult: p=0.0449*). **d**. VGlut2 density across development in layers 2-4 (left, multiple t-test, Holm sidak correction, P5: p=0.934, P15: p=0.823, P30: p=0.730, adult: p=0.106) and layers 5/6 (right, multiple t-test, P5: p=0.936, P15: p=0.700, P30: p=0.700 and adult: p=0.0565). (P5: WT: n=4mice, KO: n=7mice, P15: WT: 5mice, KO: 5mice, P30: WT: 4mice, KO: 4mice, adult: WT: 4mice, KO: 5mice; 6sections/mouse). Mean±SEM.

Supplementary Figure 4

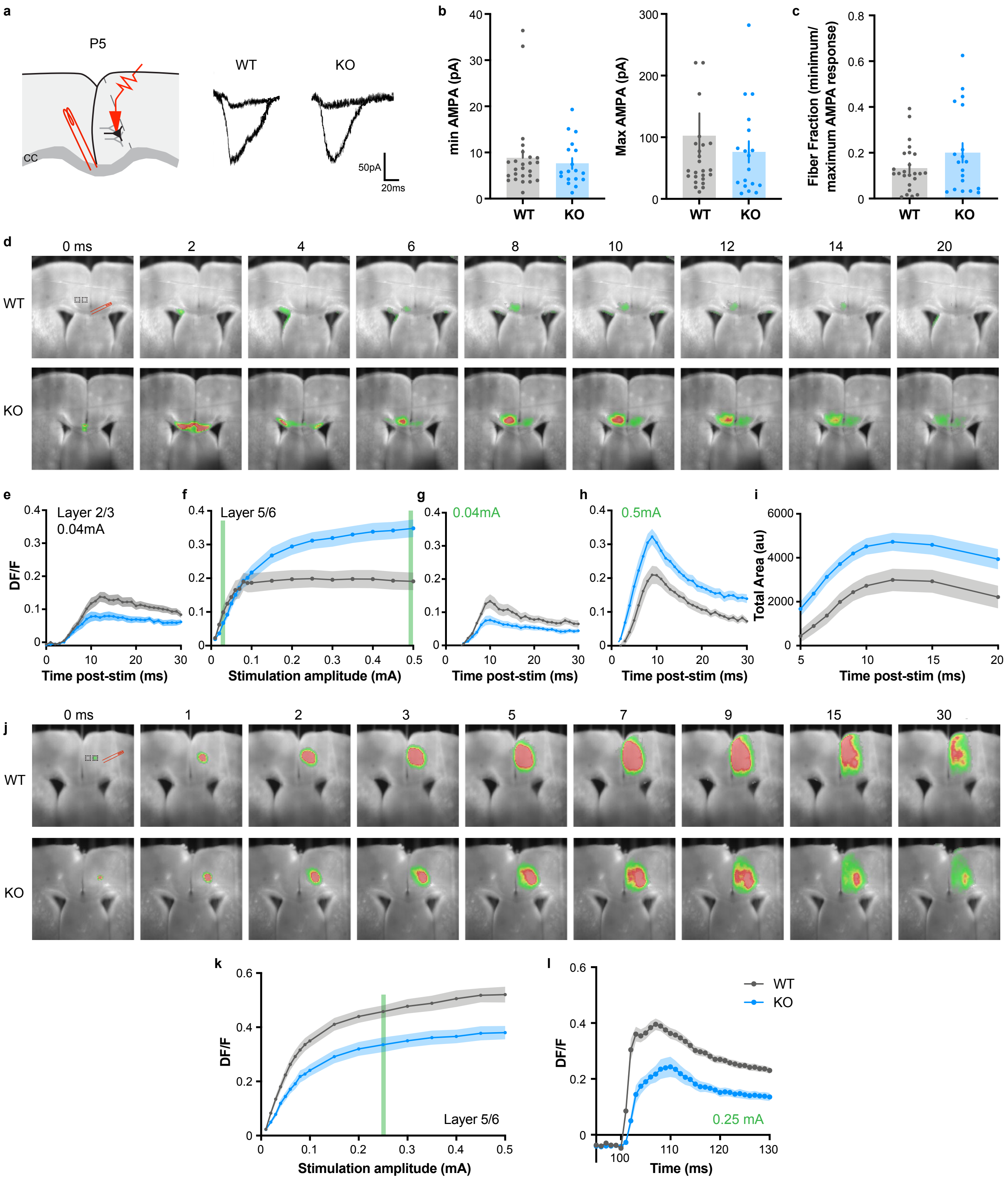

Supplementary Figure 4. Functional abnormalities of interhemispheric and local projections in the CDKL5 KO extent to deeper layers. a. Schematic of patch clamp recordings configuration at P5. **b**. Minimum eEPSC (left, WT: 7.83±1.36, n=24 cells/3mice, KO: 7.62±1.164, n=19 cells/3mice, Mann-Whitney test p=0.9449) and maximal eEPSC (right, WT: 100.1±37.29, KO: 76.36±17.24, Mann-Whitney p=0.8257). **c**. Fiber fraction representing the ratio of minimum/maximum AMPA response recorded in Pyr cells at P5 (WT: 0.13±0.02, KO: 0.20±0.04, Mann-Whitney test p=0.4061). **d**. VSDI example images of responses across time from the moment of stimulation of the corpus callosum. **e**. DF/F response over time at low stimulation (0.04mA) in layer 2/3 (Two-Way Anova, interaction genotype x time, p<0.0001***). **f**. Peak DF/F responses in L5/6 following increasing stimulation amplitude (Two-Way Anova, REML, interaction genotype x stimulation amplitude, p<0.0001***). WT: n= 11slices/8mice, KO: n= 16slices/11mice. **g-h**. DF/F responses over time in layer 5/6 at low stimulation (0.04mA, **g**, Two-Way Anova, interaction genotype x time, p<0.0001***), and high stimulation (0.5mA, **h**, Two-Way Anova, interaction genotype x time, p<0.0001***). **i**. Total area activated following corpus callosum stimulation (Two-way RM Anova, genotype p=0.0036**). **j**. VSDI example images of responses over time following stimulation of local layer 6 stimulation. **k**. Peak DF/F responses in L5/6 following increasing stimulation (Two-Way Anova, REML, interaction genotype x stimulation amplitude p<0.0001***). **l**. DF/F response over time at high stimulation in layer 5/6 (0.25mA, Mixed-effects analysis, interaction genotype x time, p<0.0001***) WT: n= 14slices/6mice, KO: n= 12slices/5mice.

Supplementary Figure 5

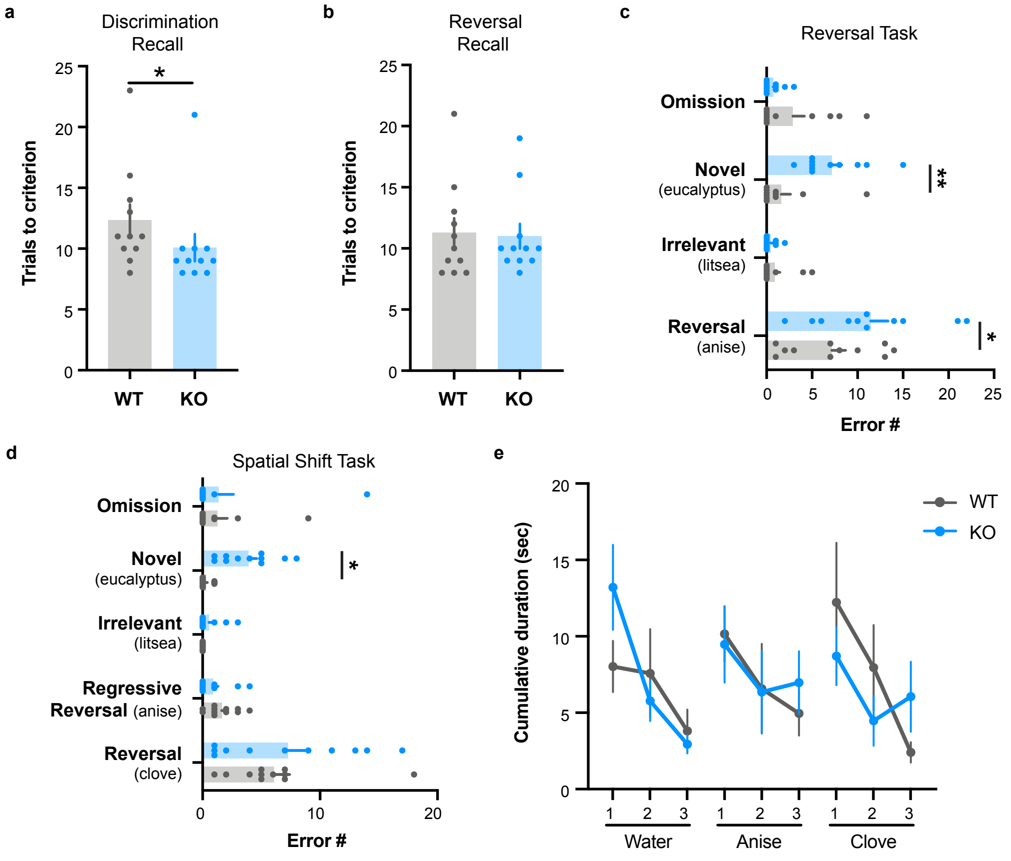

Supplementary Figure 5. Detailed findings of the four-choice foraging task and olfactory test in CDKL5 KO mice. a. Recall test 24h after the initial discrimination indicate both groups remember the task the following day and KO appear to perform even better than WT (WT: 12.36±1.27, KO: 10.09±1.12; Mann Whitney test, p=0.0231*). **b**. Both groups require the same number of trials to reach criterion in the recall test 24h after reversal task (WT: 11.27±1.19, KO: 11.00±1.02; Mann Whitney test, p=0.9352). 11 mice/genotype. **c-d.** Detailed types of error made by KO mice during the reversal task (**c**) and spatial task (**d**). Reversal-type of mistake represents the choice of odor which was previously rewarded; the irrelevant choice was to an odor never rewarded, the novel represents the new odor introduced during the reversal task, and omission represents the lack of choice. **c**. In the reversal task, CDKL5 KO mice made more perseverative errors and also explored the novel odor (Two-way Anova, interaction Genotype x type of error p=0.0239*, n=11/genotype). **d**. In the spatial shift, CDKL5 KO mice again dug more in the novel unrewarded bowl (Two-way Anova, type of error x genotype p=0.0177*). **e**. Olfactory habituation/dishabituation test in the KO mice (Two-way Anova, genotype: p=0.9869, genotype x odor interaction p=0.2612, WT: n=8mice, KO: n=11mice). Mean±SEM.

Supplementary Figure 6

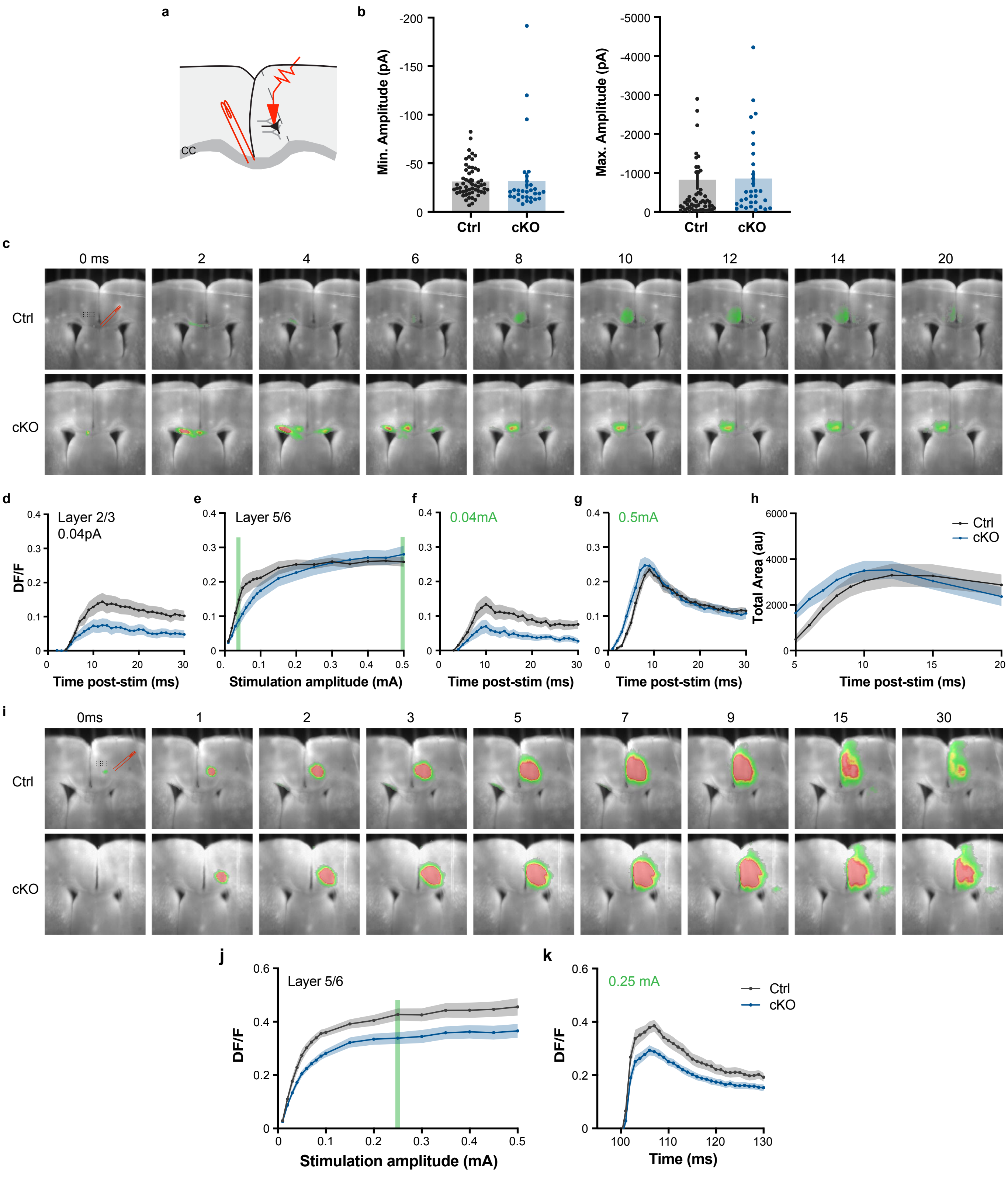

Supplementary Figure 6. Juvenile functional abnormalities following selective deletion of CDKL5 from Satb2+ neurons. a. Schematic of patch clamp recordings configuration from P15 juvenile mice. **b**. Minimum eEPSC (left, Ctrl: -31.50±2.20, n=56 cells/8mice, cKO: -31.87±6.78, n= 31cells/6mice, Mann-Whitney test p=0.0155*) and maximal eEPSC (right, Ctrl: -825.0±223.6, cKO: -852.9±182.3, Mann-Whitney p=0.0785). **c**. VSDI example images of responses across time from the moment of stimulation of the corpus callosum. **d**. DF/F response over time at low stimulation (0.04mA) in Layer 2/3 (Two-Way Anova, interaction genotype x time, p<0.0001***). **e**. Peak DF/F responses in L5/6 following increasing callosal stimulation amplitude (Two-Way Anova, REML, interaction genotype x stimulation amplitude p=0.0005***). Ctrl: n= 11slices/9mice, KO: n= 12slices/9mice. **f-g**. DF/F responses over time at low stimulation (0.04mA, **f**, Two-Way Anova, interaction genotype x time, p<0.0001***), and high stimulation (0.5mA, **g**, Two-Way Anova, interaction genotype x time, p=0.0121*). **h**. Total area activated following corpus callosum stimulation (Two-way Anova, interation time x genotype, p<0.0001***). **i**. VSDI example images of responses across time from the moment of stimulation in layer 5/6. **j**. Peak DF/F responses in L5/6 following increasing stimulation (Two-Way Anova, REML, interaction genotype x stimulation amplitude p=0.3360). **l**. DF/D response over time at high stimulation in layer 5/6 (0.25mA, Two-Way Anova, interaction genotype x time, p<0.0001***). Ctrl: n= 12slices/6mice, cKO: n= 11slices/6mice.

Supplementary Figure 7

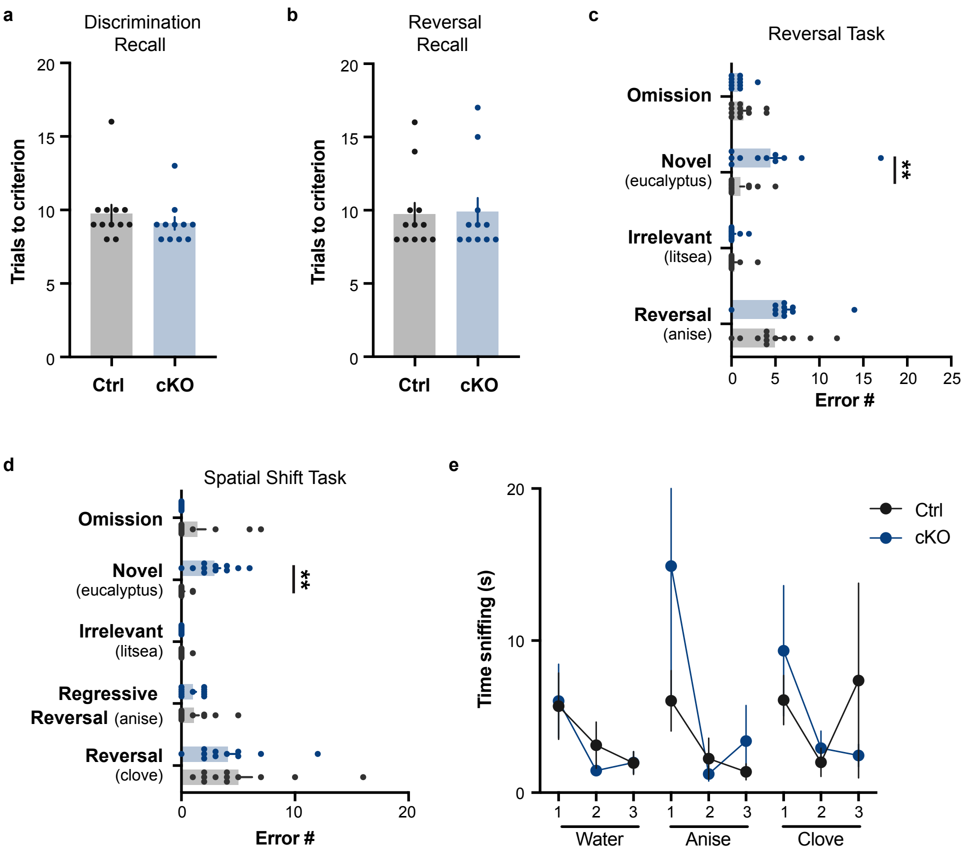

Supplementary Figure 7. Extended findings of Four-choice foraging task in cKO mice. a. Number of trials to reach criterion in the recall test 24h after the discrimination (Ctrl: 9.75±0.60, cKO: 9.09±0.43, Mann-Whitney test p=0.2288). **b**. Number of trials during recall the following day of reversal (Ctrl: 9.75±0.75, cKO: 9.91±0.94, Mann-Whitney test p=0.9118). Ctrl: n=12, cKO: n=11 mice. **c-d.** Detailed types of error made by cKO mice during the reversal task (**c**) and spatial task (**d**). **c**. In the reversal task, CDKL5 cKO mice made more errors by digging in the novel odor similar to what was found in KO mice (Two-way Anova, interaction type of errors x genotype p=0.0651, Ctrl n=12, cKO: n=11mice). **d**. Details of type of errors made in the spatial shift (Two-way Anova, interaction type of errors x genotype p=0.0043). **e**. Olfactory habituation/dishabituation test in the cKO mice shows no gross olfactory impairment (Two-way Anova, genotype: p=0.6579, genotype x odor interaction p=0.4991, Ctrl: n=13mice, cKO: n=8mice). Mean±SEM.

Supplementary Figure 8

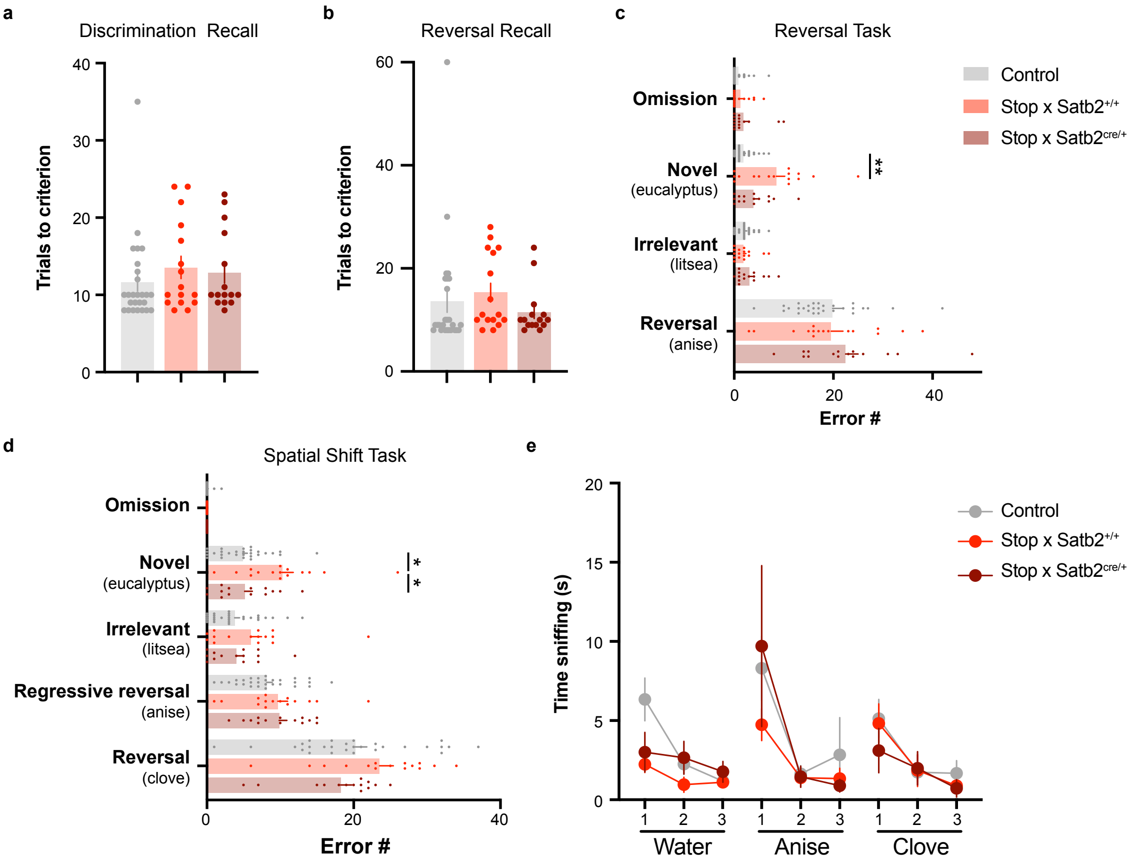

Supplementary Figure 8. Extended findings of the four-choice foraging task in CDKL5 FloxStop mice. a. Number of trials to reach criterion in the recall test 24h after the discrimination (Ctrl: 11.62±1.10, StopxSatb2$^{+/+}$: 13.31±1.46, StopxSatb2$^{cre/+}$: 13.13±1.33; One-way Anova, p=0.5543). **b**. Number of trials to reach criterion during recall of the reversal the following day (Ctrl: 13.62±2.15, StopxSatb2$^{+/+}$: 16.25±1.86, StopxSatb2$^{cre/+}$: 10.53±0.82; One-way Anova, p=0.1876). **c-d.** Types of error made by CDKL5 mutant mice during the reversal task (**c**) and spatial task (**d**). **c**. In the reversal task, the mutant mice made significantly more error digging in the novel eucalyptus bowl, as did the CDKL5 KO and cKO mice (Two-way Anova, interaction type of errors x genotype p=0.0449*) **d**. Details of type of errors made in the spatial shift (Two-way Anova, interaction type of errors x genotype p=0.0292). Ctrl: n= 26 mice, StopxSatb2$^{+/+}$: n= 16 mice, StopxSatb2$^{cre/+}$: n= 15 mice. **e**. Olfactory habituation/dishabituation test in the STOP mice show no gross olfactory impairment (Two-way Anova, genotype: p=0.1342, genotype x odor interaction p=0.8884, Ctrl: n=22 mice, StopxSatb2$^{+/+}$: n= 11mice, StopxSatb2$^{cre/+}$: n= 10mice). Mean±SEM.
