# Supplementary material for: CDKL5 sculpts functional callosal connectivity to promote cognitive flexibility
Source: Mol Psychiatry. 2023 Feb 3;29(6):1698–709. doi: 10.1038/s41380-023-01962-y (PMC11371650; doi:10.1038/s41380-023-01962-y)
Supplement: Supplementary file 2 — Supplementary material and methods [file 41380_2023_1962_MOESM2_ESM.pdf]

Materials and Methods

Animals

Animal care and experimental procedures were performed in accordance with the Institutional Animal Care and Use Committee (IACUC) of Boston Children's Hospital. CDKL5$^{-/+}$ (JAX #021967), C57BL/6J (JAX #000664), CDKL5$^{fl/fl}$ (JAX #030523) and Satb2^{Cre} (JAX #030546) breeders were purchased from Jackson Laboratories. CDKL5 FloxStop line was kindly provided by Dr. Zhaolan Zhou (UPenn) [42]. All mice were raised and bred in house on a 12hr light/dark cycle with food and water ad libitum, unless specified. Control animals of CDKL5$^{-/y}$ (KO), CDKL5$^{-/+}$ (Het), Satb2$^{cre/+}$,CDKL5$^{fl/fl}$ (cKO) and Satb2-CDKL5$^{FloxStop/y}$ mice were age- and sex-matched control littermates (CDKL5$^{+/y}$, CDKL5$^{+/+}$, Satb2$^{+/+}$,CDKL5$^{fl/fl}$ and Satb2$^{cre/+}$, CDKL5$^{+/y}$ and finally Satb2$^{+/+}$, CDKL5$^{+/y}$ and Satb2$^{cre/+}$,CDKL5$^{+/y}$ respectively). Experiments and data analysis were conducted blind to genotype but included both control and mutant per round of experiments.

Magnetic Resonance Imaging

Data acquisition

In vivo rs-fMRI data acquisition was performed in Cdkl5 mutants (KO and Het, n = 10 each) and control wild-type littermates (WT males and females, n = 10 each) using a Biospec 70/16 small animal MR system (Bruker BioSpin MRI, Ettlingen, Germany) and Paravision v6.1. Scans were obtained with a cryogenic quadrature surface coil (Bruker BioSpin AG, Fällanden, Switzerland). After common standard adjustments and anatomical images acquisition (FLASH, in-plane resolution of 0.05 × 0.02 mm^2, TE = 3.51, TR = 522 ms), a standard gradient-echo echo planar imaging sequence was used to acquire 900 volumes in 15 min (GE-EPI, repetition time TR, echo time TE = 15 ms, in-plane resolution RES = 0.22 × 0.2 mm^2, number of slice NS = 20, slice thickness ST = 0.4 mm, slice gap SG = 0.1 mm). Mild anesthesia levels were maintained using either isoflurane+medetomidine and were based on published protocols optimized for maintaining physiological stability and include intubation and mechanical ventilation [43].

Data preprocessing and analysis

Resting state fMRI datasets were preprocessed using an existing pipeline for removal of unwanted confounds from the time series as described in Zerbi et al., 2015 [43], with modifications explained in Sethi et al., 2017 [44]. Thereafter, datasets were de-spiked, band-pass filtered (0.01 - 0.25Hz), normalized first to an EPI study-specific template and then to the Allen Brain Institute reference atlas (http://mouse.brain-map.org/static/atlas) using ANTs v2.1 (http://picsl.upenn.edu/software/ants/).

BOLD time series from 38 cortical ROIs in both hemispheres were extracted using the Allen Reference Atlas ontology.

For our cortical connectivity analysis, we first calculated the cortical connectome (38x38 ROIs) in all animals by using Z-scored Pearson's correlations and we then quantified deviations between KO and WT littermates. To assess differences in connectivity profiles on a node-level, the effect size in/out of a given anatomic structure was summed. For our network analysis, we measured functional connectivity across 16 independent resting-state networks based on previous work [16]. We estimated a surrogate measure of network coupling strength using a dual regression approach [45]. Statistical analyses were then conducted using multivariate ANOVA, including Bonferroni correction implemented in SPSS24 (IBM, USA).

Ex vivo Diffusion Tensor Imaging

MRI-based diffusion-weighted (DW) imaging was carried out in Cdkl5 mutants (KO, n = 10) and control wild-type littermates (WT, n = 10) in PFA-fixed specimens as previously described [46]. Briefly, mice were deeply anesthetized with pentobarbital (100mg/kg), and cardiacally perfused using PBS followed by 4% PFA (Sigma cat# 441244). Both perfusion solutions were added with a gadolinium chelate (ProHance Bracco Diagnostics NDC 0270-1111-01) at a concentration of 10 and 1mM, respectively. Brains with cleaned skulls were then placed in Fomblin Y (Sigma-Aldrich, cat#317950-100G) and directly imaged to avoid post-extraction deformations.

DW imaging of mouse brains was performed using a 72-mm birdcage transmit coil and a saddle-shaped solenoid coil for signal reception. Each DW dataset was composed of 8 non-DW images and 81 diffusion gradient-encoding directions with $b = 3000$ s/mm^2 ($\partial = 6$ ms, $\Delta = 13$ ms) acquired using an EPI sequence with: TR/TE = 13500/27.6 ms, FOV 1.68 × 1.54 cm, matrix 120 × 110, in-plane spatial resolution 140 × 140 µm, 54 coronal slices, slice thickness 280 µm, number of averages = 20 [47].

Tract-based spatial statistics (TBSS) analysis was implemented in FSL [48]. The DW datasets were first corrected for eddy current distortions and the skull-stripped to remove extra-brain tissues. Voxelwise fractional anisotropy (FA) was then calculated, and FA maps were nonlinearly registered to an in-house FA template with FLIRT and FNIRT and thinned using a FA threshold of 0.2 to create a skeleton of the white matter. Voxelwise intergroup comparison of FA between Cdkl5 mutants and control mice was carried by using 5000 permutations ($p < 0.05$, two-tailed). FA was also regionally quantified in 3 × 3 × 1 ROI placed to probe major white matter structures, including the corpus callosum, dorsal hippocampal commissure, anterior commissure, and internal capsule. Genotype-dependent differences of regional FA were assessed

with unpaired two-tailed Student's *t* test ($q < 0.05$, two-tailed, FDR-corrected), as implemented by Prism GraphPad (v9.2).

Immunohistochemistry, imaging, and analysis

Mice were transcardially perfused with saline, followed by 4% PFA (Sigma, cat #441244). Brains were post-fixed in PFA overnight, then cryoprotected in sucrose solutions and sectioned with a cryostat (Leica CM3050 S). Free floating 35µm sections were blocked (10% normal goat serum (NGS), 0.5% Triton X-10 (Sigma)), then incubated overnight at 4°C in a primary antibody solution (5% NGS, 0.3% Triton X-10, anti-VGlut1 (Synaptic Systems cat#135 303, 1:1000), anti-Vglut2 (Synaptic Systems cat#135 404, 1:2000), anti-Myelin Basic Protein (Millipore MAB384, 1:150). Sections were then incubated in secondary antibody solution (5% NGS, 0.03% Triton-10X, goat anti-mouse Alexa 488 (Invitrogen cat#A-11001, 1:1000 dilution), goat anti-rabbit Alexa 594 (Invitrogen cat# A-11012, 1:500 dilution) in PBS). Sections were counterstained with DAPI (300nM, Invitrogen D3571) and mounted on glass slides with Vectashield mounting medium (Vector Labs, H-1200).

Images were acquired with a laser scanning confocal microscope (Zeiss 710), using the multi-channel acquisition mode. Quantitative analyses were performed on a minimum of 4-6 sections per mice in 3-6 mice per genotype. For Vglut1/2 puncta imaging, a 63x objective (1.4 NA) was used, while MBP and Vglut2 staining was imaged using a 20x objective (0.8 NA). Puncta density and intensity were measured using MacBiophotonics ImageJ software, using background subtraction, automatic thresholding, watershed and analyze particles functions to delineate individual presynaptic compartment; intensity was then measured on unaltered images and manually subtracting background intensity.

Slice preparation and electrophysiology

We evaluated fiber fraction in the corpus callosum and mEPSCs as previously described[49]. Slices were prepared from either P4-P5 or P15-P19 mice. Briefly, mice were decapitated, and the brains removed and placed in chilled oxygenated artificial cerebral spinal fluid (ACSF) containing (mM): 118NaCl, 2.5 KCl, 1.3 $MgCl_2$, 1.2 NaH_2PO_4, 2.5 $CaCl_2$, 10 glucose, and 26 $NaHCO_3$. Cortical sections were cut at 350 µm thick using a Leica VT1000S vibratome and then incubated for at least 1 h in ACSF before moving to the recording chamber. All solutions were continuously bubbled with 95% O_2–5% CO_2. Neurons were visualized using a customized Scientifica/Olympus microscope. Data was obtained with a Multiclamp 700B amplifier (Axon Instruments), digitized with Digidata 1440A (Axon Instruments) and collected with Clampex 10.7 (Axon Instruments). Recordings were filtered at 2 kHz and digitized at 10 kHz. Whole-cell

patch-clamp recordings were conducted with 4-6 MΩ pipettes. The internal solution for mEPSC recording contained (mM): 100 Cs-gluconate, 0.2 EGTA, 5 $MgCl_2$, 2 Mg ATP, 0.3 Li GTP, and 40 HEPES, pH adjusted to 7.2 with CsOH. The solution for fiber fraction recordings contained (mM): 35 CsF, 100 $CsCl_2$, 10 EGTA, and 10 HEPES, pH adjusted to 7.3 with CsOH. ACSF was supplemented during recording with 500 nM tetrodotoxin (mEPSCs only) and 50 µM picrotoxin. For all electrophysiological experiments, layer 5 pyramidal neurons in the ACC/RSP were targeted for recording. For stimulation experiments, a concentric bipolar electrode (CDKL5 KO experiments; FHC, Inc.) or a pair of glass electrodes, filled with 1 M NaCl and 25 mM HEPES (Satb2 cKO, CDKL5 Het experiments), were placed in the center of the corpus callosum and responses were evoked using a A365 Stimulus Isolator (WPI) set between 0.1-1mA. Minimal callosal responses were determined using the failure method, whereby a response had to be present in less than half of all stimulations. The maximal response was the largest evoked amplitude as defined by the response reaching a plateau for 3 consecutive increases in stimulation intensity. Fiber fraction was defined as the minimal amplitude divided by the maximum amplitude. Cells were held at −70 mV. For fiber fraction analysis, responses were analyzed with Clampfit 10.7 (Axon). Miniature EPSCs were analyzed using Minianalysis (Synaptosoft).

In vitro voltage-sensitive dye Imaging

We performed voltage-sensitive dye imaging of cingulate cortex as previously described [50]. Mice were decapitated under brief isoflurane anesthesia and the brain was rapidly removed in chilled oxygenated ACSF containing (in mM): 130 NaCl, 10 glucose, 24 $NaHCO_3$, 3.5 KCl, 1.25 NaH_2PO_4, 2.5 $CaCl_2$ and 1.5 MgCl2. Brains were sectioned (350 µm) using a Leica VT1000S vibratome. Slices were incubated for 20 min at 35 °C, and then 90 min in the voltage-sensitive dye Di-4-ANEPPS (Invitrogen; D-1199; 5 µg/ml) at RT. Either the corpus callosum bundle, or local L5/6 fibers in the cingulate cortex were stimulated (0.1-1mA, 1ms pulse) with an ACSF-filled glass pipette and delivered with a constant current stimulus isolator (Iso-Flex, A.M.P.I., new for CDKL5 female recordings) controlled by a programmable pulse generator (MiCam Ultima Software, Version 1002). Excitation light source was a LED illumination system at 530nm peak wavelength (LEX3-G, SciMedia) and reflected toward the sample by a dichroic mirror. Emitted fluorescence was long-pass filtered (590 nm) and imaged with a MiCam Ultima CMOS (complementary metal-oxide semiconductor)-based camera (SciMedia; 1-ms frame rate; 512-ms period). Fluorescent signals were integrated across regions of interest (100 × 100 µm) at a constant depth from the pia (25–150 µm from the pial surface). Time course traces were averaged across ten trials and exported to BV Ana software (SciMedia) for analysis. Fluorescence change was normalized to resting fluorescence (ΔF/F). Peak

ΔF/F was determined as the maximum average response within a time window (5–50 ms after stimulation).

Behavior Testing

Olfactory habituation/dishabituation

Olfactory discrimination was performed in a static cage, where a cotton swab was hanging from the cover of the cage. Habituation to the cage and the cotton swab lasted 30min, and then odors were dipped in the following scents: water, anise (1:100 in ddH_20) and clove (1:100 in ddH_20). Three trials of 2min each were performed, and manual scoring of the amount of time spent sniffing were recorded. These odors were selected to confirm that mice could distinguish the odors that contained the food reward in the foraging task. Mice who underwent this olfactory test did not perform the foraging task.

Four-Choice Foraging Task

Four-choice odor discrimination and reversal task were performed as previously described [51]. Briefly, the training arena consists of a clear box with four quadrants with a clear acrylic cylinder in the middle, used as the starting position for the mouse. Odor stimuli consisted of wood shavings (Kaytee aspen bedding, Chilton, WI) scented with odor cues (anise extract (McCormick, MD; clove, litsea and eucalyptus oils (1:10 in mineral oil; San Francisco Massage Supply Co., San Francisco, CA); and thymol (diluted 1:20 in 50% ethanol, Alfa Aesar, cat#A14563); mixed with shaving at 0.02ml/g). The food rewards were small pieces (~10mg) of Honey Nut Cheerios (General Mills, MN). Mice were placed on a restricted diet for 3 days prior to the start of this behavioral task (~90% weight). After a day of habituation to the arena, pots and cheerios, mice learned how to dig under the wood shaving to find the cheerio in a shaping session. In the following testing days, all pots were sham baited with a small piece of cheerio held under a mesh screen. Each task (discrimination, reversal and spatial shift) consisted in learning to distinguish between the four odors and the one associated with the buried food reward. The location of the pot with the food reward was pseudo-randomized. Mice were free to explore within a 3min trial, and the latency for the decision to dig one bowl was recorded. If no choice was made after 3min, the trial was recorded as an omission. Digging in the pot with the previously rewarded odor was a perseverative error. Irrelevant errors were the choice to dig an unrewarded odor, while the novel error was the choice of digging in the new unrewarded odor. Criterion for each task was 8 correct digging choices out of 10 consecutives trials, to a maximum of 120 trials. Two mice were unable to stay awake during the entirety of the test and were excluded from the analysis. Omission trials were analyzed separately.

Morris Water Maze

Morris water maze was run on 3-5 months old mice as previously described [52, 53]. Briefly, a pool was filled with water (26±1°C) and a white liquid tempera. The tank is subdivided into four equal quadrants, and designated with cardinal points (N, E, W and S). Mice were habituated to the arena and escape platform (0.5cm above waterline) for 2 trials. Within a trial, mice were placed in the pool for 90 sec, four times starting from each quadrant. In the training phase, mice learned the location of the submerged escape platform (NW) with the help of the visual cues placed around the arena. This training occurred over 2 days (3 and 2 trials respectively), where the starting point of immersion rotated between all four quadrants. The latency for the animal to escape onto the platform within a 90sec trial was recorded. If the time had elapsed before the mouse found the platform, it was guided there and given 5sec to orientate itself. The platform was then completely removed for the probe test, and the immersion point was furthest (SE) from the location of the expected quadrant (NW). Finally, the reversal task was run in 4 single trials where the platform was in a new quadrant (SE), and the starting point was in the opposite quadrant. All swim patterns were recorded and analyzed with the software EthoVision XT (Noldus). Mice who simply floated and did not perform the tasks were excluded from analysis.

Statistical analysis

All data are presented as mean ± standard error, with n and ages described in figure legends. Only animals/sections/slices with technical issues were excluded from analysis. Sample sizes for each in vitro and in vivo experiment was decided based on our prior work, and power analysis conducted with a false-positive rate of 0.05 and the desired power of 95%. The sample size acquired was adequately powered for making our conclusions of significant or not difference between mutants and WT mice. Each experiment included WT and mutant mice from multiple litters. Normality distribution of the data was first assessed by Shapiro-Wilk normality test. To compare between two groups, we performed the parametric unpaired two-tailed Student's *t*-test, or the nonparametric Mann-Whitney test (FDR-corrected for DTI or corrected for multiple comparison using Holm-Sidak method). Statistical tests on repeated measures were conducted using two-way ANOVA or Mixed model analyses, with Sidak correction for multiple comparisons for VSDI. Outliers were identified by the ROUT method (Q=0.1%). Statistical tests were performed using Prism (v9.4) and the probability threshold for statistical significance was set at $p<0.05$.
