# Supplementary material for: CDKL5 sculpts functional callosal connectivity to promote cognitive flexibility
Source: Mol Psychiatry. 2023 Feb 3;29(6):1698–709. doi: 10.1038/s41380-023-01962-y (PMC11371650; doi:10.1038/s41380-023-01962-y)
Supplement: Supplementary file 4 — Supplementary Statistics information [file 41380_2023_1962_MOESM4_ESM.pdf]

VSDI experiments

Multiple comparison statistical information

CDKL5 WT vs. KO males

DF/F x Stimulation intensity

Layer 2/3 (Figure 2)

Callosal stim WT vs KO

Stim intensity	p value	significance
0.01	>0.9999	ns
0.02	0.9997	ns
0.03	0.9795	ns
0.04	0.9425	ns
0.05	0.9842	ns
0.06	0.9646	ns
0.07	0.9948	ns
0.08	0.9996	ns
0.09	>0.9999	ns
0.1	>0.9999	ns
0.15	>0.9999	ns
0.2	0.892	ns
0.25	0.434	ns
0.3	0.1786	ns
0.35	0.0982	ns
0.4	0.0166	*
0.45	0.0291	*
0.5	0.0164	*

Intracortical stim WT vs. KO

Stim intensity	p value	significance
0.01	>0.9999	ns
0.02	>0.9999	ns
0.03	0.9989	ns
0.04	0.952	ns
0.05	0.7718	ns
0.06	0.6124	ns
0.07	0.5445	ns
0.08	0.4566	ns
0.09	0.3051	ns
0.1	0.1631	ns
0.15	0.012	*
0.2	0.0226	*
0.25	0.0043	**
0.3	0.0003	***
0.35	<0.0001	****
0.4	<0.0001	****
0.45	0.0002	***
0.5	<0.0001	****

DF/F x Time at 0.5 pA

Layer 2/3 (Figure 2)

Callosal stim WT vs KO

Time post-stim	p value	significance
**-5**	>0.9999	ns
**-4**	>0.9999	ns
**-3**	0.1517	ns
**-2**	>0.9999	ns
**-1**	0.6757	ns

Intracortical stim WT vs. KO

Time post-stim	p value	significance
**-5**	>0.9999	ns
**-4**	>0.9999	ns
**-3**	>0.9999	ns
**-2**	>0.9999	ns
**-1**	>0.9999	ns

0	>0.9999	ns	0	>0.9999	ns
1	>0.9999	ns	1	>0.9999	ns
2	0.4421	ns	2	>0.9999	ns
3	0.6769	ns	3	>0.9999	ns
4	0.9896	ns	4	0.8511	ns
5	0.99	ns	5	0.367	ns
6	0.7486	ns	6	0.2344	ns
7	0.3469	ns	7	0.088	ns
8	0.4355	ns	8	0.0609	ns
9	0.5089	ns	9	0.0639	ns
10	0.1989	ns	10	0.0723	ns
11	0.0818	ns	11	0.0378	*
12	0.1077	ns	12	0.0197	*
13	0.2227	ns	13	0.0229	*
14	0.2968	ns	14	0.0165	*
15	0.5327	ns	15	0.0086	**
16	0.5893	ns	16	0.0109	*
17	0.6689	ns	17	0.0073	**
18	0.8509	ns	18	0.0056	**
19	0.9248	ns	19	0.0067	**
20	0.4688	ns	20	0.0033	**
21	0.8185	ns	21	0.0069	**
22	0.5846	ns	22	0.0043	**
23	0.9645	ns	23	0.0089	**
24	0.5765	ns	24	0.0345	*
25	0.6915	ns	25	0.0125	*
26	0.4915	ns	26	0.0082	**
27	0.5415	ns	27	0.0068	**
28	0.449	ns	28	0.0078	**
29	0.7773	ns	29	0.0087	**
30	0.5472	ns	30	0.0082	**

DF/F x Stimulation intensity

Layer 5/6 (Supplementary Figure 4)

Callosal stim WT vs KO

Stim intensity	p value	significance
0.01	>0.9999	ns
0.02	>0.9999	ns
0.03	0.9992	ns
0.04	0.9991	ns

Intracortical stim WT vs. KO

Stim intensity	p value	significance
0.01	>0.9999	ns
0.02	0.9979	ns
0.03	0.7496	ns
0.04	0.5944	ns

0.05	>0.9999	ns
0.06	>0.9999	ns
0.07	>0.9999	ns
0.08	>0.9999	ns
0.09	>0.9999	ns
0.1	0.9996	ns
0.15	0.3281	ns
0.2	0.054	ns
0.25	0.0108	*
0.3	0.003	**
0.35	0.0011	**
0.4	0.0004	***
0.45	0.0001	***
0.5	<0.0001	****

0.05	0.1857	ns
0.06	0.0594	ns
0.07	0.0232	*
0.08	0.0324	*
0.09	0.0091	**
0.1	0.0093	**
0.15	0.0026	**
0.2	0.0025	**
0.25	0.0018	**
0.3	0.001	***
0.35	0.001	***
0.4	0.0002	***
0.45	0.0003	***
0.5	0.0003	***

DF/F x Time at 0.5 pA

Layer 5/6 (Supplementary Figure 4)

Callosal stim WT vs KO

Time post-stim	p value	significance
**-5**	>0.9999	ns
**-4**	>0.9999	ns
**-3**	>0.9999	ns
**-2**	>0.9999	ns
**-1**	>0.9999	ns
0	>0.9999	ns
1	>0.9999	ns
2	>0.9999	ns
3	>0.9999	ns
4	0.6267	ns
5	0.0447	*
6	0.0014	**
7	0.0009	***
8	0.0009	***
9	0.0001	***
10	0.0001	***
11	0.0027	**
12	0.0234	*
13	0.0564	ns
14	0.0228	*
15	0.0728	ns
16	0.1113	ns

Intracortical stim WT vs. KO

Time post-stim	p value	significance
**-5**	>0.9999	ns
**-4**	>0.9999	ns
**-3**	0.9966	ns
**-2**	>0.9999	ns
**-1**	>0.9999	ns
0	>0.9999	ns
1	0.0155	*
2	0.0004	***
3	0.0009	***
4	0.0016	**
5	0.0005	***
6	0.0006	***
7	0.0019	**
8	0.0311	*
9	0.0954	ns
10	0.2628	ns
11	0.0979	ns
12	0.0435	*
13	0.0216	*
14	0.0137	*
15	0.0033	**
16	0.0015	**

17	0.2306	ns	17	0.0003	***
18	0.1836	ns	18	0.0007	***
19	0.116	ns	19	0.0002	***
20	0.3167	ns	20	0.0003	***
21	0.2296	ns	21	0.0003	***
22	0.3283	ns	22	0.0002	***
23	0.21	ns	23	0.0002	***
24	0.5871	ns	24	0.0022	**
25	0.2437	ns	25	0.0004	***
26	0.5125	ns	26	0.0006	***
27	0.1638	ns	27	0.0014	**
28	0.2366	ns	28	0.0004	***
29	0.2255	ns	29	0.001	***
30	0.4305	ns	30	0.0008	***

CDKL5 Ctrl vs. cKO males

DF/F x Stimulation intensity

Layer 2/3 (Figure 4)

Callosal stim Ctrl vs cKO

Stim intensity	p value	significance
0.01	>0.9999	ns
0.02	0.9998	ns
0.03	0.9961	ns
0.04	0.8742	ns
0.05	0.7443	ns
0.06	0.8468	ns
0.07	0.9152	ns
0.08	0.9892	ns
0.09	0.9979	ns
0.1	0.9997	ns
0.15	0.9996	ns
0.2	>0.9999	ns
0.25	>0.9999	ns
0.3	>0.9999	ns
0.35	>0.9999	ns

Intracortical stim Ctrl vs. cKO

Stim intensity	p value	significance
0.01	>0.9999	ns
0.02	>0.9999	ns
0.03	>0.9999	ns
0.04	0.9999	ns
0.05	0.9561	ns
0.06	0.9883	ns
0.07	0.9913	ns
0.08	0.9947	ns
0.09	0.997	ns
0.1	0.9996	ns
0.15	>0.9999	ns
0.2	>0.9999	ns
0.25	>0.9999	ns
0.3	>0.9999	ns
0.35	>0.9999	ns

0.4	>0.9999	ns	0.4	>0.9999	ns
0.45	>0.9999	ns	0.45	>0.9999	ns
0.5	>0.9999	ns	0.5	0.9974	ns

DF/F x Time

Layer 2/3 (Figure 4)

Callosal stim Ctrl vs cKO			Intracortical stim Ctrl vs. cKO		
**Time post-stim**	**p value**	**significance**	**Time post-stim**	**p value**	**significance**
**-5**	>0.9999	ns	**-5**	0.9432	ns
**-4**	>0.9999	ns	**-4**	>0.9999	ns
**-3**	>0.9999	ns	**-3**	0.9592	ns
**-2**	>0.9999	ns	**-2**	0.383	ns
**-1**	>0.9999	ns	**-1**	0.9902	ns
0	>0.9999	ns	0	0.4385	ns
1	>0.9999	ns	1	0.6893	ns
2	>0.9999	ns	2	>0.9999	ns
3	>0.9999	ns	3	>0.9999	ns
4	>0.9999	ns	4	>0.9999	ns
5	0.7993	ns	5	>0.9999	ns
6	0.0165	*	6	>0.9999	ns
7	0.0094	**	7	>0.9999	ns
8	0.4411	ns	8	>0.9999	ns
9	0.9974	ns	9	>0.9999	ns
10	0.9999	ns	10	>0.9999	ns
11	>0.9999	ns	11	>0.9999	ns
12	>0.9999	ns	12	>0.9999	ns
13	>0.9999	ns	13	>0.9999	ns
14	>0.9999	ns	14	>0.9999	ns
15	>0.9999	ns	15	>0.9999	ns
16	0.998	ns	16	>0.9999	ns
17	0.9983	ns	17	>0.9999	ns
18	0.9824	ns	18	>0.9999	ns
19	>0.9999	ns	19	>0.9999	ns
20	0.9956	ns	20	>0.9999	ns
21	0.9956	ns	21	>0.9999	ns
22	0.9543	ns	22	>0.9999	ns
23	0.9486	ns	23	>0.9999	ns
24	>0.9999	ns	24	>0.9999	ns
25	>0.9999	ns	25	>0.9999	ns
26	0.9991	ns	26	>0.9999	ns
27	0.9993	ns	27	>0.9999	ns
28	>0.9999	ns	28	>0.9999	ns

29	>0.9999	ns	29	>0.9999	ns
30	>0.9999	ns	30	>0.9999	ns

DF/F x Stimulation intensity

Layer 5/6 (Supplementary Figure 6)

Callosal stim Ctrl vs cKO

Stim intensity	p value	significance
0.01	>0.9999	ns
0.02	>0.9999	ns
0.03	0.9796	ns
0.04	0.5392	ns
0.05	0.1593	ns
0.06	0.2872	ns
0.07	0.4847	ns
0.08	0.7029	ns
0.09	0.915	ns
0.1	0.9804	ns
0.15	0.998	ns
0.2	>0.9999	ns
0.25	>0.9999	ns
0.3	>0.9999	ns
0.35	>0.9999	ns
0.4	>0.9999	ns
0.45	>0.9999	ns
0.5	>0.9999	ns

Intracortical stim Ctrl vs. cKO

Stim intensity	p value	significance
0.01	>0.9999	ns
0.02	0.9961	ns
0.03	0.6145	ns
0.04	0.2103	ns
0.05	0.0734	ns
0.06	0.045	*
0.07	0.0308	*
0.08	0.0313	*
0.09	0.0284	*
0.1	0.0646	ns
0.15	0.2085	ns
0.2	0.292	ns
0.25	0.0331	*
0.3	0.0888	ns
0.35	0.0776	ns
0.4	0.1022	ns
0.45	0.0482	*
0.5	0.0204	*

DF/F x Time

Layer 5/6 (Supplementary Figure 6)

Callosal stim Ctrl vs cKO

Time post-stim	p value	significance
**-5**	>0.9999	ns
**-4**	>0.9999	ns
**-3**	>0.9999	ns
**-2**	>0.9999	ns
**-1**	>0.9999	ns
0	>0.9999	ns
1	>0.9999	ns

Intracortical stim Ctrl vs. cKO

Time post-stim	p value	significance
**-5**	0.9964	ns
**-4**	0.9964	ns
**-3**	0.999	ns
**-2**	0.9981	ns
**-1**	0.911	ns
0	0.9944	ns
1	0.9342	ns

2	>0.9999	ns	2	0.9184	ns
3	>0.9999	ns	3	0.8065	ns
4	0.8229	ns	4	0.561	ns
5	0.3237	ns	5	0.4472	ns
6	0.0556	ns	6	0.3	ns
7	0.5998	ns	7	0.0706	ns
8	0.2901	ns	8	0.2209	ns
9	0.9979	ns	9	0.313	ns
10	>0.9999	ns	10	0.423	ns
11	>0.9999	ns	11	0.5764	ns
12	>0.9999	ns	12	0.4238	ns
13	>0.9999	ns	13	0.4607	ns
14	>0.9999	ns	14	0.6185	ns
15	>0.9999	ns	15	0.7515	ns
16	>0.9999	ns	16	0.793	ns
17	>0.9999	ns	17	0.7176	ns
18	>0.9999	ns	18	0.6463	ns
19	>0.9999	ns	19	0.8658	ns
20	>0.9999	ns	20	0.8611	ns
21	>0.9999	ns	21	0.8742	ns
22	>0.9999	ns	22	0.7518	ns
23	>0.9999	ns	23	0.9604	ns
24	>0.9999	ns	24	0.8003	ns
25	>0.9999	ns	25	0.6552	ns
26	>0.9999	ns	26	0.9552	ns
27	>0.9999	ns	27	0.9664	ns
28	>0.9999	ns	28	0.9315	ns
29	>0.9999	ns	29	0.592	ns
30	>0.9999	ns	30	0.9245	ns

CDKL5 WT vs. Het females

DF/F x Stimulation intensity

Layer 2/3 (Supplementary Figure 2)

Callosal stim WT females vs Het females

Stim intensity	p value	significance
0.01	>0.9999	ns
0.03	>0.9999	ns
0.05	0.9999	ns

Intracortical stim WT females vs. Het females

Stim intensity	p value	significance
0.01	0.9761	ns
0.03	0.5587	ns
0.05	0.5321	ns

0.07	>0.9999	ns
0.09	0.9935	ns
0.1	0.9434	ns
0.25	0.9913	ns
0.5	0.1416	ns
0.75	0.0156	*
1	0.0002	***

0.07	0.7418	ns
0.09	0.7929	ns
0.1	0.7652	ns
0.25	0.7123	ns
0.5	0.9987	ns
0.75	0.9737	ns

DF/F x Time

Layer 2/3 (Supplementary Figure 2)

Callosal stim WT females vs Het females

Time post-stim	p value	significance
**-5**	>0.9999	ns
**-4**	>0.9999	ns
**-3**	>0.9999	ns
**-2**	>0.9999	ns
**-1**	>0.9999	ns
0	>0.9999	ns
1	>0.9999	ns
2	0.4545	ns
3	0.102	ns
4	<0.0001	****
5	<0.0001	****
6	<0.0001	****
7	<0.0001	****
8	<0.0001	****
9	<0.0001	****
10	<0.0001	****
11	<0.0001	****
12	<0.0001	****
13	0.0001	***
14	0.0099	**
15	0.195	ns
16	0.3628	ns
17	0.9894	ns
18	0.9535	ns
19	>0.9999	ns
20	>0.9999	ns
21	>0.9999	ns
22	>0.9999	ns

Intracortical stim WT females vs. Het females

Time post-stim	p value	significance
**-5**	>0.9999	ns
**-4**	>0.9999	ns
**-3**	>0.9999	ns
**-2**	>0.9999	ns
**-1**	>0.9999	ns
0	>0.9999	ns
1	>0.9999	ns
2	>0.9999	ns
3	>0.9999	ns
4	>0.9999	ns
5	>0.9999	ns
6	>0.9999	ns
7	>0.9999	ns
8	>0.9999	ns
9	>0.9999	ns
10	>0.9999	ns
11	>0.9999	ns
12	>0.9999	ns
13	>0.9999	ns
14	>0.9999	ns
15	>0.9999	ns
16	>0.9999	ns
17	>0.9999	ns
18	>0.9999	ns
19	>0.9999	ns
20	>0.9999	ns
21	>0.9999	ns
22	>0.9999	ns

23	>0.9999	ns	23	>0.9999	ns
24	>0.9999	ns	24	>0.9999	ns
25	>0.9999	ns	25	>0.9999	ns
26	>0.9999	ns	26	>0.9999	ns
27	>0.9999	ns	27	>0.9999	ns
28	>0.9999	ns	28	>0.9999	ns
29	0.9998	ns	29	>0.9999	ns
30	>0.9999	ns	30	>0.9999	ns

DF/F x Stimulation intensity

Layer 5/6 (Supplementary Figure 2)

Callosal stim WT females vs Het females

Stim intensity	p value	significance
0.01	>0.9999	ns
0.03	>0.9999	ns
0.05	0.5891	ns
0.07	0.8604	ns
0.09	0.9681	ns
0.1	0.9613	ns
0.25	>0.9999	ns
0.5	0.4502	ns
0.75	0.0766	ns
1	0.1324	ns

Intracortical stim WT females vs. Het females

Stim intensity	p value	significance
0.01	0.8824	ns
0.03	0.8383	ns
0.05	0.3759	ns
0.07	0.7712	ns
0.09	0.8927	ns
0.1	0.8302	ns
0.25	>0.9999	ns
0.5	0.9619	ns
0.75	0.1772	ns

DF/F x Time

Layer 5/6 (Supplementary Figure 2)

Callosal stim WT females vs Het females

Time post-stim	p value	significance
**-5**	>0.9999	ns
**-4**	>0.9999	ns
**-3**	>0.9999	ns
**-2**	>0.9999	ns
**-1**	>0.9999	ns
0	>0.9999	ns

Intracortical stim WT females vs. Het females

Time post-stim	p value	significance
**-5**	>0.9999	ns
**-4**	>0.9999	ns
**-3**	>0.9999	ns
**-2**	>0.9999	ns
**-1**	>0.9999	ns
0	>0.9999	ns

1	>0.9999	ns	1	0.9991	ns
2	>0.9999	ns	2	>0.9999	ns
3	0.8556	ns	3	>0.9999	ns
4	0.009	**	4	>0.9999	ns
5	<0.0001	****	5	>0.9999	ns
6	<0.0001	****	6	>0.9999	ns
7	<0.0001	****	7	>0.9999	ns
8	<0.0001	****	8	>0.9999	ns
9	<0.0001	****	9	>0.9999	ns
10	<0.0001	****	10	>0.9999	ns
11	<0.0001	****	11	>0.9999	ns
12	0.0226	*	12	>0.9999	ns
13	0.0024	**	13	>0.9999	ns
14	0.9562	ns	14	>0.9999	ns
15	0.153	ns	15	>0.9999	ns
16	0.2897	ns	16	>0.9999	ns
17	0.9956	ns	17	>0.9999	ns
18	0.9993	ns	18	>0.9999	ns
19	0.9655	ns	19	>0.9999	ns
20	0.9989	ns	20	>0.9999	ns
21	0.9998	ns	21	>0.9999	ns
22	0.7233	ns	22	>0.9999	ns
23	0.8987	ns	23	>0.9999	ns
24	0.9159	ns	24	>0.9999	ns
25	0.0738	ns	25	>0.9999	ns
26	0.8654	ns	26	>0.9999	ns
27	0.9031	ns	27	>0.9999	ns
28	0.7393	ns	28	>0.9999	ns
29	0.5134	ns	29	>0.9999	ns
30	0.1047	ns	30	>0.9999	ns

Behavior CDKL5 FloxStop x Satb2 cre (Figure 6)

MWM - Learning phase

Trial 1		
Control vs. CDKL5 FS X Satb2 cre -	0.2774	ns
Control vs. CDKL5 FS X Satb2 cre +	0.6407	ns
CDKL5 FS X Satb2 cre - vs. CDKL5 FS X Satb2 cre +	0.0808	ns
Trial 2		
Control vs. CDKL5 FS X Satb2 cre -	0.02	*
Control vs. CDKL5 FS X Satb2 cre +	0.9965	ns
CDKL5 FS X Satb2 cre - vs. CDKL5 FS X Satb2 cre +	0.0191	*

Trial 3

Control vs. CDKL5 FS X Satb2 cre -	0.0032	**
Control vs. CDKL5 FS X Satb2 cre +	0.9888	ns
CDKL5 FS X Satb2 cre - vs. CDKL5 FS X Satb2 cre +	0.0056	**

Trial 4

Control vs. CDKL5 FS X Satb2 cre -	0.0066	**
Control vs. CDKL5 FS X Satb2 cre +	0.8102	ns
CDKL5 FS X Satb2 cre - vs. CDKL5 FS X Satb2 cre +	0.0489	*

Trial 5

Control vs. CDKL5 FS X Satb2 cre -	0.0055	**
Control vs. CDKL5 FS X Satb2 cre +	0.8804	ns
CDKL5 FS X Satb2 cre - vs. CDKL5 FS X Satb2 cre +	0.0158	*
